# Dynamic rough set learning for reliable early warning in industrial time-series systems

**DOI:** 10.1038/s41598-026-62429-y

**Published:** 2026-07-21

**Authors:** Amr Zakaria

**Affiliations:** Department of Mathematics, Faculty of Education, Ain Shams University, Cairo, 11341 Egypt

**Keywords:** Rough sets, Dynamic rough approximations, Time-series classification, Early warning systems, Boundary region, Feature reduction, Industrial fault diagnosis, Explainable artificial intelligence, Engineering, Mathematics and computing

## Abstract

Industrial early-warning systems require models that can detect transitional risk states before failure while also explaining uncertainty in the resulting decisions. Existing machine-learning and deep-learning approaches can achieve strong predictive performance, but they often provide limited information about whether a time-series window is certainly normal, certainly critical or only ambiguously classified. This paper addresses this problem by proposing a dynamic rough set learning framework for uncertainty-aware early warning in industrial time-series systems. The proposed method converts multivariate sensor trajectories into sliding-window decision systems and constructs time-dependent neighborhood rough approximations for evolving decision classes. A rough early-warning index is introduced to quantify boundary-region expansion over time, and a dynamic dependency-based feature-reduction procedure is developed to retain informative window descriptors while preserving rough decision ability. Unlike purely black-box classifiers, the framework provides class predictions together with support gaps, local uncertainty scores, boundary-region information, deferred decisions and warning rates. The framework is evaluated on the NASA C-MAPSS FD001 turbofan degradation benchmark. The full dynamic rough set model achieves a test accuracy of 0.8647, balanced accuracy of 0.7570 and Macro-F1 score of 0.7895. Random Forest and XGBoost obtain stronger pure classification scores, with Macro-F1 values of 0.8715 and 0.8611, respectively. However, the proposed method supplies additional rough uncertainty outputs that are useful for inspecting transitional and ambiguous windows. Dynamic rough feature reduction decreases the feature set from 118 to 40 features while retaining a test accuracy of 0.8459 and Macro-F1 score of 0.7564. Robustness analysis further shows that the model is stable under moderate Gaussian feature noise, whereas high missingness mainly affects the warning class. These findings indicate that the proposed framework is most suitable for early-warning settings where interpretability, uncertainty qualification and inspection prioritization are as important as raw classification accuracy.

## Introduction

Industrial time-series monitoring is an important task in condition-based maintenance, fault diagnosis and reliability engineering. Modern machines, engines and production systems generate multivariate sensor streams that describe operating conditions, degradation patterns and possible abnormal behavior. In these settings, the practical goal is not only to classify a system after a fault has occurred, but also to identify warning signs while the system is still in a transitional state.

Early warning is challenging because the transition from normal behavior to critical behavior is usually gradual and uncertain. Sensor signals may be noisy, incomplete, nonstationary or affected by unit-specific operating variability. Consequently, a model that assigns every window to a single crisp label may hide the uncertainty of borderline cases. This is particularly important in industrial decision support, where an ambiguous window may not be a simple error; it may indicate that additional inspection, monitoring or preventive maintenance is required.

Machine-learning and deep-learning methods have been widely used for time-series classification, forecasting and anomaly detection. Convolutional, recurrent, graph-based and hybrid architectures can learn expressive temporal representations and often achieve strong predictive performance. However, many such models provide limited information about the distinction between certain, possible and ambiguous decisions. In safety-critical early-warning applications, this limitation is important because maintenance engineers may need to know not only the predicted class, but also whether the prediction is well supported or uncertain.

Rough set theory provides a natural mathematical framework for representing uncertainty through lower approximations, upper approximations and boundary regions. The lower approximation describes objects that certainly belong to a decision class, while the upper approximation describes objects that possibly belong to it. The difference between these two regions is the boundary region, which represents insufficiently determined cases. This three-region interpretation is closely related to three-way decision making and is well suited to early-warning systems, where a boundary case may be deferred for further inspection instead of being forced into a final class.

Despite this advantage, many rough-set models are formulated for static information systems. Industrial time-series data, by contrast, evolve over time and must be analyzed through windows, trajectories and changing local neighborhoods. This creates the need for a dynamic rough set formulation that can follow how uncertainty evolves across sequential decision systems. The present paper addresses this need by developing a dynamic rough set learning framework for reliable early warning in industrial time-series systems.

The proposed framework transforms multivariate time series into sliding-window decision systems, constructs time-dependent neighborhood rough approximations, and uses the evolution of boundary regions as an early-warning signal. It also introduces a rough early-warning index and a dynamic rough feature-reduction procedure. The resulting model is not intended to replace all high-accuracy black-box classifiers. Rather, it is designed to provide an interpretable uncertainty-aware layer that can support inspection prioritization, deferred decisions and maintenance decision making.

The main contributions of this paper are as follows.

(i) A dynamic rough set framework is proposed for time-series early warning by replacing a single static information table with a sequence of sliding-window decision systems.
(ii) Time-dependent neighborhood rough approximations are constructed to represent certain, possible and boundary regions of evolving decision classes.
(iii) A rough early-warning index is introduced to quantify the relative expansion of the boundary region over time.
(iv) A dynamic dependency-based feature-reduction procedure is developed to select informative time-series descriptors while preserving rough decision ability.
(v) A complete reproducible algorithmic pipeline is provided, including window construction, feature extraction, normalization, dynamic rough approximation, feature reduction, validation-based threshold selection, classification and warning generation.
(vi) The method is evaluated on the NASA C-MAPSS FD001 turbofan degradation benchmark and is compared with standard machine-learning baselines in terms of classification performance and uncertainty-aware outputs.

The novelty of the proposed framework lies in combining dynamic sliding-window decision systems with rough boundary-region monitoring. The model does not merely apply a static rough classifier to precomputed features. Instead, it follows the temporal behavior of rough approximations and uses boundary expansion, support gaps and local uncertainty as interpretable warning indicators. This makes the framework particularly useful in problems where uncertain transitional states are operationally important.

The rest of the paper is organized as follows. “Preliminaries” presents the mathematical preliminaries and related literature. “Dynamic rough set model for time-series systems” introduces the dynamic rough set model for time-series systems. “Dynamic rough feature reduction” develops the dynamic rough feature-reduction method. “Algorithmic framework” describes the complete algorithmic framework. “Experimental study” presents the experimental study. “Results” reports the results. “Discussion” discusses interpretability, robustness and practical implications. “Limitations” states the limitations, and “Conclusion” concludes the paper.

## Preliminaries

This section collects the mathematical background used in the paper and positions the proposed framework within the related literature. The purpose is not to claim the standard rough-set properties as new results, but to fix notation and make the later dynamic construction self-contained. Whenever a property follows directly from classical rough sets, neighborhood rough sets, three-way decisions or rough-set feature reduction, the corresponding standard references are cited explicitly.

The following subsections recall the basic static information system, decision system, classical indiscernibility relation and rough approximations. These notions are then extended toward neighborhood, temporal and feature-reduction settings.

For clarity and consistency, Abbreviation section summarizes the main notation used throughout the paper.

### Classical rough decision systems

Classical rough-set analysis starts from an information system

1 $$\begin{aligned} IS=(U,A,V,f), \end{aligned}$$

where *U* is a finite universe of objects, *A* is a finite set of attributes, $V=\bigcup _{a\in A}V_a$ is the set of attribute values and $f:U\times A\rightarrow V$ is an information function. If a decision attribute $d\notin A$ is added, then one obtains the decision system

2 $$\begin{aligned} DS=(U,A\cup \{d\},V,f). \end{aligned}$$

For $B\subseteq A$, the classical indiscernibility relation is

3 $$\begin{aligned} x\equiv _B y \quad \Longleftrightarrow \quad f(x,a)=f(y,a)\quad \text {for all }a\in B. \end{aligned}$$

The equivalence class of *x* with respect to $\equiv _B$ is denoted by $[x]_B$. For a decision class $D\subseteq U$, the classical lower and upper approximations are

4 $$\begin{aligned} \underline{B}(D)&=\{x\in U:[x]_B\subseteq D\},\nonumber \\ \overline{B}(D)&=\{x\in U:[x]_B\cap D\ne \emptyset \}. \end{aligned}$$

The corresponding boundary region is

5 $$\begin{aligned} BND_B(D)=\overline{B}(D)\setminus \underline{B}(D). \end{aligned}$$

Rough set theory was introduced by Pawlak as a mathematical approach for reasoning with vagueness, uncertainty and indiscernibility^1^. Its central idea is that a concept may not be exactly definable from the available attributes. Instead, it is represented through a lower approximation, containing objects that certainly belong to the concept, and an upper approximation, containing objects that possibly belong to it. The difference between these two regions is the boundary region, already defined in (5). This boundary is not a computational artifact; it is the mathematical representation of insufficient information^1–3^.

Let $DS=(U,A\cup \{d\},V,f)$ be the decision system defined in (2). The decision attribute *d* induces a decision partition

6 $$\begin{aligned} U/d=\{D_1,D_2,\ldots ,D_m\}, \end{aligned}$$

where each $D_i\subseteq U$ is a decision class. For a set of condition attributes $B\subseteq A$, the positive region of *d* with respect to *B* is defined by

7 $$\begin{aligned} POS_B(d)=\bigcup _{D_i\in U/d}\underline{B}(D_i). \end{aligned}$$

Thus, $POS_B(d)$ contains all objects that can be certainly assigned to one of the decision classes using the attributes in *B*. The corresponding dependency degree is

8 $$\begin{aligned} \gamma _B(d)=\frac{|POS_B(d)|}{|U|}. \end{aligned}$$

The value $\gamma _B(d)\in [0,1]$ measures how much of the decision structure is preserved by the attribute subset *B*. This quantity is a standard basis for rough-set feature reduction and reduct construction^1,4,5^.

#### Definition 1

Let $DS=(U,A\cup \{d\},V,f)$ be a decision system. A subset $B\subseteq A$ is called a reduct of *A* with respect to *d* if

9 $$\begin{aligned} \gamma _B(d)=\gamma _A(d) \end{aligned}$$

and no proper subset $C\subsetneq B$ satisfies $\gamma _C(d)=\gamma _A(d)$.

The reduct in Definition 1 preserves the classification ability of the full attribute set while removing redundant attributes. Recent surveys continue to emphasize rough feature selection as an active research direction, especially for high-dimensional, incomplete, weakly supervised and multi-granularity data^5,6^. In the present paper, the static criterion in (9) will be replaced by a temporal criterion because the objects are not fixed records, but evolving time-series windows.

### Neighborhood rough approximations

The classical rough-set model uses equivalence classes. This is suitable when attributes are categorical and exact equality is meaningful. However, industrial time-series systems usually generate numerical features, such as signal amplitude, entropy, energy, frequency-domain measures and degradation indicators. For such data, exact equality is too restrictive. Neighborhood rough sets replace equivalence classes by metric or tolerance neighborhoods^7–9^.

Let $B\subseteq A$. For each attribute $a\in A$, assume that

$$\begin{aligned} \delta _a:V_a\times V_a\rightarrow [0,1] \end{aligned}$$

is a normalized local dissimilarity. Let ${\bf w}=(w_a)_{a\in A}$ be a nonnegative weight vector satisfying

10 $$\begin{aligned} 0\le w_a\le 1\quad (a\in A), \qquad \sum _{a\in A}w_a=1. \end{aligned}$$

The weighted dissimilarity induced by *B* is defined by

11 $$\begin{aligned} \delta _B(x,y)=\sum _{a\in B}w_a\,\delta _a(f(x,a),f(y,a)), \qquad x,y\in U. \end{aligned}$$

For a tolerance radius $\rho \ge 0$, the neighborhood granule of $x\in U$ is

12 $$\begin{aligned} N_B^\rho (x)=\{y\in U:\delta _B(x,y)\le \rho \}. \end{aligned}$$

Using (12), the neighborhood lower and upper approximations of $D\subseteq U$ are defined by

13 $$\begin{aligned} \begin{aligned} \underline{N}_B^\rho (D)&=\{x\in U:N_B^\rho (x)\subseteq D\},\\ \overline{N}_B^\rho (D)&=\{x\in U:N_B^\rho (x)\cap D\ne \emptyset \}. \end{aligned} \end{aligned}$$

The nonemptiness of neighborhood granules is a standard consequence of the definition of neighborhood rough sets. In particular, if $\delta _a(u,u)=0$ for every $a\in A$ and $u\in V_a$, then

14 $$\begin{aligned} x\in N_B^\rho (x) \end{aligned}$$

for every $B\subseteq A$, $\rho \ge 0$, and $x\in U$. Hence every neighborhood granule is nonempty^7,8^. This property will be used later without further proof.

Neighborhood rough sets are particularly relevant to the present paper because the objects are sliding windows represented by continuous features. Recent work has proposed adaptive neighborhood rough-set models for hybrid numerical and categorical data^8^, while weighted *k*-nearest-neighbor rough-set models have been developed to reduce the sensitivity of neighborhood rough sets to noise^9^. These developments support the use of adaptive and distance-based granules in the proposed dynamic model.

Rough-set models have also been extended in uncertain decision-making environments beyond ordinary classification. Recent large-group decision-making studies combine rough approximations with cloud models in order to represent linguistic uncertainty, granularity and randomness. For example, interval rough integrated cloud models have been used for large group decision-making under uncertain evaluation information^10^. More recently, rough integrated asymmetric cloud models have been developed for multi-granularity linguistic large-group decision-making^11^. Although these works address group decision-making rather than industrial time-series early warning, they confirm the broader usefulness of rough-set extensions for handling uncertainty, granularity and incomplete decisiveness. The present paper follows this general direction, but applies rough uncertainty modeling to dynamic sliding-window time-series decision systems.

### Three-way decisions and the meaning of the boundary region

The boundary region in rough set theory has a natural decision interpretation. Three-way decision theory formalizes this idea by replacing forced binary decisions with three possible actions: acceptance, rejection and deferment^2,3^. For a decision class $D\subseteq U$, the three regions induced by a rough approximation are

15 $$\begin{aligned} \begin{aligned} POS_B(D)&=\underline{B}(D),\\ BND_B(D)&=\overline{B}(D)\setminus \underline{B}(D),\\ NEG_B(D)&=U\setminus \overline{B}(D). \end{aligned} \end{aligned}$$

In this interpretation, the positive, negative and boundary regions represent certain acceptance, rejection and deferment, respectively. The boundary region therefore contains the objects for which the available information is insufficient.

For early-warning systems, the boundary region has a direct practical meaning. A time-series window in the boundary region is neither certainly normal nor certainly abnormal. It represents an intermediate state that may require further observation, expert inspection or preventive intervention. Recent temporal three-way decision models have been used in emergency admission prediction by combining multigranulation neighborhood rough sets with hidden Markov modeling^12^. The present work is related to this temporal view, but it develops a different mechanism: instead of estimating hidden state transitions, it measures the expansion of rough boundary regions over sliding-window decision systems.

#### Remark 2

The proposed model does not treat the boundary region as an error set. Rather, the boundary region is treated as a meaningful uncertainty layer. This distinction is important for safety-critical industrial monitoring applications, where uncertain cases may be more informative than incorrectly forced labels.

### Time-series windows as dynamic decision systems

Let

$$\begin{aligned} \mathcal {X}^{(i)}=({\bf x}_1^{(i)},{\bf x}_2^{(i)},\ldots ,{\bf x}_{L_i}^{(i)}) \end{aligned}$$

be the *i*-th multivariate time series, where ${\bf x}_\ell ^{(i)}\in \mathbb {R}^p$. For a fixed window length $h\in \mathbb {N}$ and stride $s\in \mathbb {N}$, define the *t*-th window of the *i*-th time series by

16 $$\begin{aligned} W_{i,t}= ({\bf x}_{1+(t-1)s}^{(i)},{\bf x}_{2+(t-1)s}^{(i)},\ldots , {\bf x}_{h+(t-1)s}^{(i)}), \end{aligned}$$

whenever the indices are well defined. Each window is mapped into a feature vector by a feature map

17 $$\begin{aligned} \psi (W_{i,t})= (\psi _1(W_{i,t}),\psi _2(W_{i,t}),\ldots ,\psi _r(W_{i,t}))\in \mathbb {R}^r. \end{aligned}$$

The components of $\psi$ may include statistical, temporal, spectral and complexity-based descriptors. Typical examples include mean, variance, root mean square, skewness, kurtosis, entropy, peak-to-peak value, spectral energy, dominant frequency and wavelet energy. Such hand-crafted features remain useful in condition monitoring because they provide interpretable and computationally efficient summaries of signal dynamics^13^.

For each time index *t*, let

18 $$\begin{aligned} U_t=\{u_{i,t}: i\in I_t\} \end{aligned}$$

be the finite set of windows available at time *t*, where $u_{i,t}$ denotes the object associated with $W_{i,t}$. The corresponding time-dependent decision system is

19 $$\begin{aligned} DS_t=(U_t,A\cup \{d\},V_t,f_t), \end{aligned}$$

where $A=\{a_1,a_2,\ldots ,a_r\}$ is the set of extracted features, and

20 $$\begin{aligned} f_t(u_{i,t},a_j)=\psi _j(W_{i,t}), \qquad j=1,2,\ldots ,r. \end{aligned}$$

The decision attribute *d* may represent normality, abnormality, fault type, degradation state, remaining-useful-life category or an early-warning label.

The construction in (19) is the key transition from static rough-set analysis to dynamic rough-set learning. Instead of approximating decision classes in a single table, the proposed method approximates decision classes across a sequence of tables indexed by time. This is consistent with recent interest in time-series learning, where deep-learning surveys emphasize the importance of automatic representation learning for sequential classification and regression^14^, and graph-based time-series surveys emphasize the role of temporal and inter-variable dependence^15^. However, the present paper uses rough approximations rather than black-box temporal representations.

Recent work on information granules for time-series classification has also shown that consecutive signal windows can be transformed into compact summaries that improve interpretability and robustness^16^. The present model shares the window-based viewpoint, but differs by constructing lower, upper and boundary approximations from the window granules.

### Dynamic neighborhood relations

Because the objects in $U_t$ are numerical feature vectors, the time-dependent version of (11) is used. For $B\subseteq A$, define

21 $$\begin{aligned} \delta _{B,t}(x,y) = \sum _{a\in B}w_{a,t}\, \delta _{a,t}(f_t(x,a),f_t(y,a)), \qquad x,y\in U_t, \end{aligned}$$

where

22 $$\begin{aligned} 0\le w_{a,t}\le 1, \qquad \sum _{a\in A}w_{a,t}=1. \end{aligned}$$

The time dependence in (21) allows the model to adapt to changing signal regimes. For example, feature importance may differ between early degradation, near-failure behavior and normal operation.

For a tolerance radius $\rho _t\ge 0$, define the dynamic neighborhood relation $R_{B,t}^{\rho _t}$ by

23 $$\begin{aligned} x\,R_{B,t}^{\rho _t}\,y \quad \Longleftrightarrow \quad \delta _{B,t}(x,y)\le \rho _t. \end{aligned}$$

The associated dynamic neighborhood granule is

24 $$\begin{aligned} {[}x]_{B,t}^{\rho _t} = \{y\in U_t:x\,R_{B,t}^{\rho _t}\,y\}. \end{aligned}$$

For a decision class $D\subseteq U_t$, the dynamic lower and upper approximations are

25 $$\begin{aligned} \begin{aligned} \underline{R}_{B,t}^{\rho _t}(D)&= \{x\in U_t:[x]_{B,t}^{\rho _t}\subseteq D\},\\ \overline{R}_{B,t}^{\rho _t}(D)&= \{x\in U_t:[x]_{B,t}^{\rho _t}\cap D\ne \emptyset \}. \end{aligned} \end{aligned}$$

The corresponding dynamic boundary region is

26 $$\begin{aligned} BND_{B,t}^{\rho _t}(D) = \overline{R}_{B,t}^{\rho _t}(D) \setminus \underline{R}_{B,t}^{\rho _t}(D). \end{aligned}$$

The monotonicity of neighborhood approximations with respect to the tolerance radius is standard in neighborhood rough-set models^7–9^. More precisely, if $0\le \rho _1\le \rho _2$, then

27 $$\begin{aligned} {[}x]_{B,t}^{\rho _1}\subseteq [x]_{B,t}^{\rho _2}. \end{aligned}$$

Consequently, for every $D\subseteq U_t$,

28 $$\begin{aligned} \begin{aligned} \underline{R}_{B,t}^{\rho _2}(D)&\subseteq \underline{R}_{B,t}^{\rho _1}(D),\\ \overline{R}_{B,t}^{\rho _1}(D)&\subseteq \overline{R}_{B,t}^{\rho _2}(D). \end{aligned} \end{aligned}$$

Thus, increasing the tolerance radius makes certain membership more restrictive and possible membership less restrictive. This observation is important for the interpretation of the dynamic boundary region. The choice of $\rho _t$ therefore directly affects the balance between certainty and uncertainty.

### Rough early-warning index

The proposed early-warning mechanism is based on the time evolution of the boundary region. For $D\subseteq U_t$, define

29 $$\begin{aligned} E_{B,t}^{\rho _t}(D) = {\left\{ \begin{array}{ll} 1-\dfrac{|\underline{R}_{B,t}^{\rho _t}(D)|}{|\overline{R}_{B,t}^{\rho _t}(D)|}, & \overline{R}_{B,t}^{\rho _t}(D)\ne \emptyset ,\\ 0, & \overline{R}_{B,t}^{\rho _t}(D)=\emptyset . \end{array}\right. } \end{aligned}$$

The index in (29) is the proposed dynamic rough early-warning index, with explicit dependence on *B* and $\rho _t$. It measures the relative gap between possible membership and certain membership.

Since the lower approximation is contained in the upper approximation, the index in (29) satisfies

30 $$\begin{aligned} 0\le E_{B,t}^{\rho _t}(D)\le 1. \end{aligned}$$

This follows directly from the standard rough-set inclusion

$$\begin{aligned} \underline{R}_{B,t}^{\rho _t}(D)\subseteq \overline{R}_{B,t}^{\rho _t}(D), \end{aligned}$$

which is inherited from classical and neighborhood rough approximations^1,2,7^. Therefore, (29) can be interpreted as a normalized uncertainty measure.

A value close to zero means that most possible members of *D* are also certain members. A value close to one means that the upper approximation is much larger than the lower approximation, and therefore the decision class contains a large uncertain component. In an early-warning context, the sequence

$$\begin{aligned} E_{B,1}^{\rho _1}(D), E_{B,2}^{\rho _2}(D), \ldots , E_{B,T}^{\rho _T}(D) \end{aligned}$$

is interpreted as an uncertainty trajectory. A persistent increase may indicate that the system is moving toward an unstable or abnormal state.

### Dynamic dependency degree and temporal reducts

Feature reduction is one of the most important applications of rough set theory. In the static case, the dependency degree $\gamma _B(d)$ in (8) measures the classification power of an attribute subset. For dynamic time-series systems, this idea must be evaluated across time.

For $B\subseteq A$, define the dynamic positive region by

31 $$\begin{aligned} POS_{B,t}^{\rho _t}(d) = \bigcup _{D_i\in U_t/d} \underline{R}_{B,t}^{\rho _t}(D_i). \end{aligned}$$

The dynamic dependency degree is

32 $$\begin{aligned} \gamma _{B,t}^{\rho _t}(d) = \frac{|POS_{B,t}^{\rho _t}(d)|}{|U_t|}. \end{aligned}$$

To measure the average decision ability of *B* over a time horizon $\{1,2,\ldots ,T\}$, define

33 $$\begin{aligned} \Gamma _B(d) = \frac{1}{T} \sum _{t=1}^{T} \gamma _{B,t}^{\rho _t}(d). \end{aligned}$$

The exact equality $\Gamma _B(d)=\Gamma _A(d)$ may be too strict in noisy time-series systems. Therefore, a tolerance parameter $\eta \ge 0$ is introduced.

#### Definition 3

Let $\eta \ge 0$. A subset $B\subseteq A$ is called an $\eta$-temporal reduct of *A* with respect to *d* if

34 $$\begin{aligned} \Gamma _A(d)-\Gamma _B(d)\le \eta \end{aligned}$$

and no proper subset $C\subsetneq B$ satisfies

$$\begin{aligned} \Gamma _A(d)-\Gamma _C(d)\le \eta . \end{aligned}$$

The temporal reduct in Definition 3 generalizes the static reduct in Definition 1. It selects features that preserve decision ability over the sequence of dynamic decision systems, rather than at only one time point. This is important for early-warning applications because a feature may be weak in normal periods but highly informative near an abnormal event.

By construction,

35 $$\begin{aligned} 0\le \gamma _{B,t}^{\rho _t}(d)\le 1, \qquad 0\le \Gamma _B(d)\le 1. \end{aligned}$$

These bounds follow from the standard definition of the positive region and dependency degree in rough-set-based feature reduction^1,4,5^. The temporal quantity $\Gamma _B(d)$ in (33) is the arithmetic mean of dynamic dependency degrees, and therefore remains in the same interval.

### Sequential industrial forecasting and imbalanced learning

The proposed method is motivated not only by rough set theory, but also by the broader literature on industrial sequential forecasting and decision support. In aviation applications, aircraft trajectory and fuel-consumption prediction have been studied using covariance bidirectional extreme learning machines, showing that sequential learning models can support operational planning in aircraft systems^17^. In manufacturing, smoothing and matrix-decomposition-based stacked bidirectional GRU models have been proposed for machine downtime forecasting, where the smoothing and decomposition stages are used to improve robustness under noisy and abnormal time-series data^18^. Beyond maintenance and aviation, sequential learning has also been used in resource and supply-chain related forecasting; for example, multi-echelon tandem learning combines decomposition, convolutional, recurrent and attention components for iron-ore price forecasting^19^. These studies show the general importance of dynamic, noisy and sequential industrial data, even when the application domains differ from turbofan degradation monitoring.

The present paper is related to these works at the level of problem structure: all of them address sequential data whose future behavior is operationally important. However, the proposed method differs in its mathematical objective. It does not primarily seek a more complex neural architecture. Instead, it aims to represent the evolving uncertainty of a decision class by lower approximations, upper approximations and boundary regions. This distinction is important because an early-warning system may need to justify why a window is considered ambiguous, rather than only return a numerical score.

Class imbalance is another practical issue in early-warning and degradation problems, because transition or warning states are often less frequent than normal states. Imbalance-aware learning methods, such as balanced weighted extreme learning machines, show that class-frequency effects can strongly influence predictive performance in risk and productivity problems^20^. In the present framework, imbalance is addressed empirically by using balanced accuracy, Macro-F1 and warning-class F1 in model assessment, and methodologically by allowing validation-based thresholds that are selected with attention to the warning class. For severely imbalanced applications, the rough support score in (62) can also be replaced by the class-balanced support

36 $$\begin{aligned} \pi ^\textrm{bal}_{c,t}(x) = \frac{ \dfrac{|[x]_{B,t}^{\rho _t}\cap D_{c,t}|}{|D_{c,t}|+\varepsilon } }{ \sum _j \dfrac{|[x]_{B,t}^{\rho _t}\cap D_{j,t}|}{|D_{j,t}|+\varepsilon } }, \qquad \varepsilon >0, \end{aligned}$$

which normalizes local evidence by the size of each decision class. This option reduces majority-class dominance while retaining the rough-neighborhood interpretation.

### Position of the present framework

The proposed framework is related to, but different from, several recent directions. It is related to neighborhood rough sets because it uses tolerance-based granules for numerical data^7–9^. It is related to three-way decision theory because the boundary region is interpreted as a meaningful deferred-decision region^2,3,12^. It is related to time-series classification because sliding windows are transformed into feature-based objects, as in many industrial signal-processing pipelines^13–15^. It is also related to information granulation for time-series classification because it summarizes local windows into interpretable objects^16^.

However, the present paper differs from these works in its central objective. It does not only classify time-series windows. It constructs a sequence of rough approximation spaces and studies the temporal behavior of their boundary regions. The rough early-warning index in (29) is therefore not a conventional classifier score. It is an uncertainty measure derived from the evolving gap between lower and upper approximations.

Compared with the fuzzy cognitive map model in^21^, the present framework uses dynamic neighborhood rough approximations rather than fuzzy causal maps, adaptive fuzzy weights or expert-edited edge weights. Its main target is early warning in industrial time-series systems.

The definitions and cited standard properties in this section will be used as follows. The dynamic decision system in (19) is the basic data representation. The dynamic approximations in (25) define certain, possible and uncertain windows. The boundary region in (26) supports the early-warning index in (29). Finally, the dynamic dependency degree in (32) and the temporal reduct in Definition 3 provide the feature-reduction component of the proposed method.

## Dynamic rough set model for time-series systems

This section introduces the proposed dynamic rough set model for time-series systems. The model is built on the preliminaries of “Preliminaries”, especially the dynamic decision system in (19), the dynamic neighborhood granule in (24), the dynamic approximations in (25), and the rough early-warning index in (29). The purpose of the present section is to turn these ingredients into a complete learning framework for industrial time-series early warning.

The main idea is simple. A time series is first divided into consecutive or overlapping windows. Each window is represented by an interpretable feature vector. At each time step, these windows form a time-dependent decision system. A neighborhood rough approximation is then constructed for each decision class. The lower approximation represents windows that can be assigned to a decision class with certainty, the upper approximation represents windows that may belong to the class, and the boundary region represents uncertain windows. The temporal behavior of the boundary region is then used as an early-warning signal.

### Dynamic representation and preprocessing

Let

$$\begin{aligned} \mathcal {X}^{(i)}=({\bf x}_1^{(i)},{\bf x}_2^{(i)},\ldots ,{\bf x}_{L_i}^{(i)}) \end{aligned}$$

be the *i*-th multivariate time series, where ${\bf x}_\ell ^{(i)}\in \mathbb {R}^p$. In industrial applications, $\mathcal {X}^{(i)}$ may represent a vibration signal, acoustic measurement, sensor stream or degradation trajectory. The proposed framework uses the sliding-window representation already defined in (16). Each window $W_{i,t}$ is mapped into a feature vector through the feature map $\psi$ in (17). This feature-based representation is widely used in interpretable signal monitoring and remains important even in the presence of deep-learning alternatives^13,14^.

The proposed dynamic rough set learning procedure consists of the following stages:

(i) construct sliding windows from each time series;
(ii) extract interpretable statistical, temporal, spectral and complexity-based features;
(iii) normalize the extracted features using only the training partition;
(iv) construct the dynamic decision system $DS_t$ in (19);
(v) define a time-dependent neighborhood relation using an adaptive radius;
(vi) compute the lower, upper and boundary approximations of each decision class;
(vii) evaluate the rough early-warning index in (29);
(viii) generate a decision according to the rough region and the temporal behavior of the warning index.

This construction extends the static rough-set view of Pawlak^1^ and the neighborhood rough-set view of Hu et al.^7^ to a sequence of time-indexed approximation spaces. It is also connected to three-way decision theory because the boundary region is interpreted as a deferred or uncertain decision region rather than as a classification error^2,3^.

#### Window-level feature representation

For each time series $\mathcal {X}^{(i)}$, let $h\in \mathbb {N}$ be the window length and $s\in \mathbb {N}$ be the stride. The window $W_{i,t}$ is defined in (16). Its ending time index is

37 $$\begin{aligned} e_{i,t}=h+(t-1)s. \end{aligned}$$

The feature map $\psi$ in (17) transforms $W_{i,t}$ into an *r*-dimensional vector. In the proposed framework, the feature set is decomposed into four groups:

38 $$\begin{aligned} A=A_\textrm{stat}\cup A_\textrm{temp}\cup A_\textrm{spec}\cup A_\textrm{comp}, \end{aligned}$$

Here the four groups are statistical, temporal, spectral and complexity descriptors.

Typical statistical features include

39 $$\begin{aligned} \begin{aligned} \mu (W)&=\frac{1}{h}\sum _{\ell =1}^{h}x_\ell ,\\ \sigma ^2(W)&=\frac{1}{h-1}\sum _{\ell =1}^{h}\bigl (x_\ell -\mu (W)\bigr )^2,\\ \textrm{RMS}(W)&=\Big (\frac{1}{h}\sum _{\ell =1}^{h}x_\ell ^2\Big )^{1/2}. \end{aligned} \end{aligned}$$

Other useful features include skewness, kurtosis, peak-to-peak amplitude, crest factor and interquartile range. For a univariate signal window $W=(x_1,\ldots ,x_h)$, the peak-to-peak amplitude is

40 $$\begin{aligned} \textrm{P2P}(W)=\max _{1\le \ell \le h}x_\ell -\min _{1\le \ell \le h}x_\ell . \end{aligned}$$

A simple temporal trend feature is the least-squares slope

41 $$\begin{aligned} \textrm{Slope}(W)= \frac{\sum _{\ell =1}^{h}(\ell -\bar{\ell })(x_\ell -\mu (W))}{\sum _{\ell =1}^{h}(\ell -\bar{\ell })^2}, \qquad \bar{\ell }=\frac{h+1}{2}. \end{aligned}$$

Frequency-domain features may be computed from the discrete Fourier transform. If $\widehat{x}_k$ denotes the discrete Fourier coefficient of *W*, then the normalized spectral energy over a frequency band $\mathcal {F}$ is

42 $$\begin{aligned} \textrm{SE}_{\mathcal {F}}(W)= \frac{\sum _{k\in \mathcal {F}}|\widehat{x}_k|^2}{\sum _{k}|\widehat{x}_k|^2+\varepsilon }. \end{aligned}$$

Here, $\varepsilon >0$ is a small numerical constant used only to avoid division by zero. Complexity features may include entropy-type quantities. For example, if $p_1,\ldots ,p_q$ are normalized histogram frequencies extracted from *W*, then the Shannon entropy feature is

43 $$\begin{aligned} H(W)=-\sum _{j=1}^{q}p_j\log (p_j+\varepsilon ). \end{aligned}$$

For multivariate signals, the same features may be computed channel-wise and then concatenated. If ${\bf x}_\ell =(x_{\ell ,1},\ldots ,x_{\ell ,p})$, then

44 $$\begin{aligned} \psi (W_{i,t}) = \bigl (\psi ^{(1)}(W_{i,t}),\psi ^{(2)}(W_{i,t}),\ldots ,\psi ^{(p)}(W_{i,t}),\psi ^{(\textrm{cross})}(W_{i,t})\bigr ), \end{aligned}$$

where $\psi ^{(q)}$ denotes features extracted from the *q*-th channel and $\psi ^{(\textrm{cross})}$ may include cross-channel correlation, covariance or coherence features.

#### Normalization and leakage control

To avoid data leakage, all normalization parameters must be estimated from the training data only. Let $\mathcal {T}_\textrm{train}$ denote the set of training windows. For each feature $a_j\in A$, define

45 $$\begin{aligned} m_j=\min _{u_{i,t}\in \mathcal {T}_\textrm{train}} f_t(u_{i,t},a_j), \qquad M_j=\max _{u_{i,t}\in \mathcal {T}_\textrm{train}} f_t(u_{i,t},a_j). \end{aligned}$$

The normalized feature value is

46 $$\begin{aligned} \widetilde{f}_t(u_{i,t},a_j) = \frac{f_t(u_{i,t},a_j)-m_j}{M_j-m_j+\varepsilon }. \end{aligned}$$

The same values $m_j$ and $M_j$ are then used for validation and test windows. This is essential in industrial evaluation because using information from future or test windows would artificially improve performance.

After normalization, the dynamic information function in (20) is replaced by

47 $$\begin{aligned} \widetilde{f}_t(u_{i,t},a_j)=\psi _j^\textrm{norm}(W_{i,t}), \qquad j=1,2,\ldots ,r. \end{aligned}$$

For simplicity, the notation $f_t$ will be used again for the normalized information function in the rest of the paper.

#### Decision labels and early-warning horizons

The decision attribute *d* depends on the application. In ordinary time-series classification, *d* may be the class label supplied by the dataset. In early-warning applications, the decision should also encode whether a window occurs before an abnormal event. Suppose that the *i*-th time series has an event time $\tau _i$, such as the occurrence of a machine fault, the onset of an alarm condition, or the end of useful life of an engine. Let $\Delta \in \mathbb {N}$ be a warning horizon. The warning label may be defined by

48 $$\begin{aligned} d_\Delta (u_{i,t})= {\left\{ \begin{array}{ll} 0, & e_{i,t}<\tau _i-\Delta ,\\ 1, & \tau _i-\Delta \le e_{i,t}<\tau _i,\\ 2, & e_{i,t}\ge \tau _i,\\ \end{array}\right. } \end{aligned}$$

where 0 denotes normal behavior, 1 denotes a pre-event warning state, and 2 denotes an abnormal or post-event state. In datasets where only binary labels are available, the decision attribute may be simplified to

49 $$\begin{aligned} d(u_{i,t})= {\left\{ \begin{array}{ll} 0, & \text {normal window},\\ 1, & \text {abnormal or high-risk window}. \end{array}\right. } \end{aligned}$$

The formulation in (48) is more informative for early-warning analysis, while (49) is useful when event-time annotation is unavailable.

For each time *t*, the decision attribute induces the partition

50 $$\begin{aligned} U_t/d=\{D_{1,t},D_{2,t},\ldots ,D_{m_t,t}\}, \end{aligned}$$

where each $D_{c,t}$ contains windows whose decision label is *c*. In binary detection, $m_t=2$ whenever both classes are present. In warning-horizon detection, $m_t\le 3$, depending on which labels appear in $U_t$.

### Dynamic neighborhoods and rough approximations

The dynamic neighborhood relation in (23) depends on the dissimilarity $\delta _{B,t}$ and the tolerance radius $\rho _t$. In practical time-series systems, the same fixed radius may be unsuitable across all windows because signal variability may change over time. Therefore, the proposed model uses an adaptive radius selected from the empirical distribution of pairwise distances.

For $$B\subseteq A$$, let

51 $$\begin{aligned} \mathcal {D}_{B,t} = \{\delta _{B,t}(x,y):x,y\in U_t,\ x\ne y\}. \end{aligned}$$

For a parameter $q_\rho \in (0,1)$, define

52 $$\begin{aligned} \rho _{B,t}= Q_{q_\rho }(\mathcal {D}_{B,t}), \end{aligned}$$

where $Q_{q_\rho }$ is the empirical $q_\rho$-quantile. Small values of $q_\rho$ generate smaller neighborhoods and therefore stricter granules, while larger values generate wider neighborhoods. This quantile-based strategy is consistent with the adaptive spirit of recent neighborhood rough-set methods for numerical and hybrid data^8,9^.

In some applications, abrupt changes in $\rho _{B,t}$ may create unstable approximation regions. To stabilize the radius, one may use exponential smoothing:

53 $$\begin{aligned} \bar{\rho }_{B,t} = \lambda _\rho \rho _{B,t} + (1-\lambda _\rho )\bar{\rho }_{B,t-1}, \qquad 0<\lambda _\rho \le 1, \end{aligned}$$

with $\bar{\rho }_{B,1}=\rho _{B,1}$. The smoothed radius $\bar{\rho }_{B,t}$ can then replace $\rho _t$ in (23). When no smoothing is needed, one simply takes $\bar{\rho }_{B,t}=\rho _{B,t}$.

#### Dynamic rough approximation of decision classes

For each *t*, each attribute subset $B\subseteq A$, and each decision class $D_{c,t}\in U_t/d$, the dynamic lower approximation, upper approximation and boundary region are computed using (25) and (26). To simplify notation in this section, write

54 $$\begin{aligned} \underline{R}_{B,t}(D_{c,t}) = \underline{R}_{B,t}^{\rho _t}(D_{c,t}), \qquad \overline{R}_{B,t}(D_{c,t}) = \overline{R}_{B,t}^{\rho _t}(D_{c,t}), \end{aligned}$$

and

55 $$\begin{aligned} BND_{B,t}(D_{c,t}) = BND_{B,t}^{\rho _t}(D_{c,t}). \end{aligned}$$

The three rough regions have the following interpretation:

(i) $x\in \underline{R}_{B,t}(D_{c,t})$ means that all windows similar to *x* belong to class *c*;
(ii) $x\in \overline{R}_{B,t}(D_{c,t})$ means that at least one window similar to *x* belongs to class *c*;
(iii) $x\in BND_{B,t}(D_{c,t})$ means that *x* is compatible with class *c*, but not certainly assigned to it.

This is the dynamic counterpart of the classical certain–possible–uncertain interpretation of rough sets^1–3^.

For binary early-warning detection, let $D_{1,t}$ denote the abnormal or high-risk class. The dynamic rough regions of $D_{1,t}$ then have direct operational meaning:

(i) $\underline{R}_{B,t}(D_{1,t})$: windows that are certainly high-risk;
(ii) $BND_{B,t}(D_{1,t})$: windows that are uncertain or transitional;
(iii) $U_t\setminus \overline{R}_{B,t}(D_{1,t})$: windows that are certainly not high-risk.

#### Temporal rough uncertainty and early-warning score

The rough early-warning index in (29) is applied to the target warning or abnormal class. For binary detection, define

56 $$\begin{aligned} E_t^+=E_{B,t}^{\rho _t}(D_{1,t}). \end{aligned}$$

For multi-class warning labels as in (48), one may focus on the pre-event and abnormal classes:

57 $$\begin{aligned} E_t^\textrm{risk} = \max \{E_{B,t}^{\rho _t}(D_{1,t}),E_{B,t}^{\rho _t}(D_{2,t})\}, \end{aligned}$$

whenever both classes are present. Alternatively, a weighted risk index may be used:

58 $$\begin{aligned} E_t^\textrm{risk} = \sum _{c\in \mathcal {C}_\textrm{risk}}\alpha _c E_{B,t}^{\rho _t}(D_{c,t}), \qquad \alpha _c\ge 0,\quad \sum _{c\in \mathcal {C}_\textrm{risk}}\alpha _c=1, \end{aligned}$$

where $\mathcal {C}_\textrm{risk}$ is the set of labels considered operationally risky.

The value of $E_t^\textrm{risk}$ measures the current rough uncertainty of the risk-related classes. However, early warning also depends on the temporal change of this uncertainty. Therefore, define the first-order change

59 $$\begin{aligned} \Delta E_t^\textrm{risk} = E_t^\textrm{risk}-E_{t-1}^\textrm{risk}, \qquad t\ge 2, \end{aligned}$$

and the moving average

60 $$\begin{aligned} \overline{E}_{t,L}^\textrm{risk} = \frac{1}{L} \sum _{j=0}^{L-1}E_{t-j}^\textrm{risk}, \qquad t\ge L. \end{aligned}$$

The moving average reduces the effect of isolated noisy windows. A persistent increase in $\overline{E}_{t,L}^\textrm{risk}$ indicates that the system is spending more time in uncertain rough regions, which may precede an abnormal event.

##### Definition 4

Let $\theta _E\in [0,1]$, $\theta _\Delta \ge 0$, and $L\in \mathbb {N}$. A time $t\ge L$ is called a dynamic rough warning time if

61 $$\begin{aligned} \overline{E}_{t,L}^\textrm{risk}\ge \theta _E \quad \text {or} \quad \Delta E_t^\textrm{risk}\ge \theta _\Delta . \end{aligned}$$

The thresholds $\theta _E$ and $\theta _\Delta$ should be selected on the validation set. They should not be tuned on the test set.

### Decision rule, parameters and interpretation

The proposed framework produces two types of outputs: a rough decision and an early-warning signal. For a test window $x\in U_t$, its rough membership status with respect to a decision class $D_{c,t}$ is determined by its dynamic neighborhood $[x]_{B,t}^{\rho _t}$. Define

62 $$\begin{aligned} \pi _{c,t}(x) = \frac{|[x]_{B,t}^{\rho _t}\cap D_{c,t}|}{|[x]_{B,t}^{\rho _t}|}. \end{aligned}$$

The quantity $\pi _{c,t}(x)$ is not introduced as a probability model. It is a neighborhood-based rough support score measuring the proportion of neighbors of *x* that belong to class *c*.

The predicted class is

63 $$\begin{aligned} \widehat{d}_t(x)= \arg \max _{c}\pi _{c,t}(x). \end{aligned}$$

The confidence gap is

64 $$\begin{aligned} G_t(x)= \pi _{\widehat{d}_t(x),t}(x) - \max _{c\ne \widehat{d}_t(x)}\pi _{c,t}(x). \end{aligned}$$

For a threshold $\theta _G\in [0,1]$, the rough decision is given by

65 $$\begin{aligned} \textrm{Decision}_t(x)= {\left\{ \begin{array}{ll} \textrm{accept}\ \widehat{d}_t(x),& G_t(x)\ge \theta _G \ \text {and}\ x\notin BND_{B,t}(D_{\widehat{d}_t(x),t}),\\ \textrm{defer},& G_t(x)<\theta _G \ \text {or}\ x\in BND_{B,t}(D_{\widehat{d}_t(x),t}). \end{array}\right. } \end{aligned}$$

Thus, a window is confidently classified only when the local rough support is sufficiently separated from competing classes and the window is not located in the boundary region of its predicted class. This decision mechanism follows the three-way interpretation of rough approximations, where uncertain cases are deferred rather than forced into a possibly unreliable class^2,3,12^.

For early-warning applications, the final warning output is

66 $$\begin{aligned} \textrm{Warning}_t= {\left\{ \begin{array}{ll} 1,& \textrm{Decision}_t(x)=\textrm{defer}\ \text {and}\ E_t^\textrm{risk}\ge \theta _E,\\ 1,& \Delta E_t^\textrm{risk}\ge \theta _\Delta ,\\ 0,& \text {otherwise}.\\ \end{array}\right. } \end{aligned}$$

This rule combines local uncertainty of the current window with global temporal uncertainty of the risk-related class.

#### Model parameters

The proposed dynamic rough set model contains the following parameters:

$$\begin{aligned} h,\quad s,\quad B,\quad {\bf w}_t,\quad q_\rho ,\quad \lambda _\rho ,\quad L,\quad \theta _E,\quad \theta _\Delta ,\quad \theta _G. \end{aligned}$$

Their roles are summarized as follows:

(i) *h* controls the amount of temporal information contained in each window;
(ii) *s* controls the overlap between successive windows;
(iii) $B\subseteq A$ is the selected feature subset;
(iv) ${\bf w}_t=(w_{a,t})_{a\in A}$ controls the relative importance of features at time *t*;
(v) $q_\rho$ determines the quantile used to construct the adaptive neighborhood radius;
(vi) $\lambda _\rho$ controls the temporal smoothing of the radius;
(vii) *L* is the moving-average length for the risk index;
(viii) $\theta _E$ is the warning threshold for the rough early-warning index;
(ix) $\theta _\Delta$ is the threshold for the increase in rough uncertainty;
(x) $\theta _G$ is the local confidence-gap threshold.

All parameters must be selected using the training and validation partitions only. The test partition is reserved for final evaluation. This separation is important for reproducibility and for avoiding overly optimistic early-warning performance.

#### Complete dynamic rough set model

The complete model is denoted by

67 $$\begin{aligned} \mathfrak {D} = \bigl ( \{DS_t\}_{t=1}^{T}, B, \{\delta _{B,t}\}_{t=1}^{T}, \{\rho _t\}_{t=1}^{T}, \{\underline{R}_{B,t},\overline{R}_{B,t},BND_{B,t}\}_{t=1}^{T}, \{E_t^\textrm{risk}\}_{t=1}^{T} \bigr ). \end{aligned}$$

The first component $\{DS_t\}_{t=1}^{T}$ represents the evolving time-indexed decision systems. The second component *B* is the selected feature subset. The dissimilarities and radii define the dynamic neighborhood structure. The lower, upper and boundary approximations define the rough decision regions. Finally, the sequence $\{E_t^\textrm{risk}\}_{t=1}^{T}$ represents the temporal uncertainty trajectory used for early warning.

**Algorithm 1:**
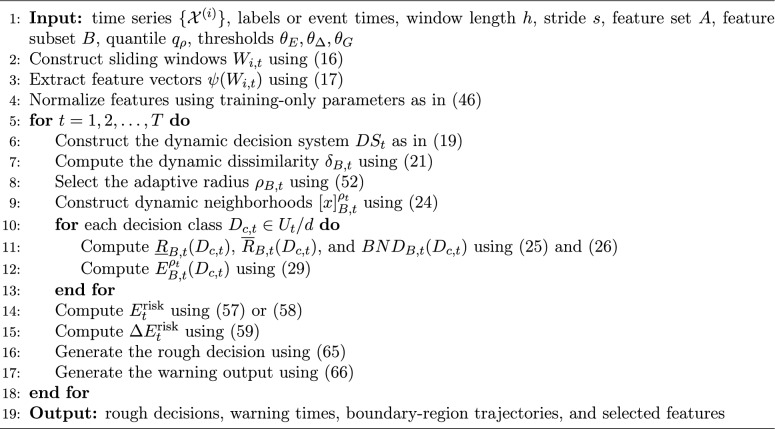
Dynamic rough set model for time-series early warning.

The output of Algorithm 1 is not limited to a predicted label. It also includes the boundary-region trajectory and the rough early-warning trajectory. These outputs are central to the interpretability of the proposed approach. They allow one to distinguish between confidently normal windows, confidently abnormal windows and uncertain windows that may require further monitoring.

#### Interpretation of the model

The proposed dynamic rough set model has three levels of interpretation. The first level is the feature level, where each window is represented by interpretable time-series descriptors. The second level is the rough-region level, where each decision class is described by lower, upper and boundary approximations. The third level is the temporal level, where the evolution of the boundary region is summarized by the rough early-warning index.

This multi-level interpretation is important for industrial monitoring applications. A boundary-region window may correspond to an early degradation stage in which vibration, acoustic or sensor features are beginning to deviate from normal patterns, but are not yet sufficiently abnormal for a confident automatic alarm. The model therefore does not hide uncertainty inside a single hard label. Instead, it makes uncertainty explicit through rough regions and uses its temporal evolution as an early-warning signal.

## Dynamic rough feature reduction

Feature reduction is a central component of rough-set-based decision analysis. In the classical setting, a reduct is an attribute subset that preserves the dependency degree of the full condition-attribute set, as recalled in Definition 1. In the present paper, however, the objects are time-series windows and the decision system changes with time. Therefore, feature reduction should not be performed only once on a single static table. It should preserve the decision ability of the dynamic rough approximation system across the time horizon under consideration.

This section develops a dynamic rough feature-reduction procedure for the proposed model. The method uses the dynamic positive region in (31), the dynamic dependency degree in (32), and the temporal dependency degree in (33). The goal is to select a compact subset of time-series features that preserves the evolving decision structure while reducing redundancy and improving interpretability. This follows the general rough-set philosophy of dependency-preserving feature reduction^1,4,5^, but adapts it to sliding-window time-series systems.

### Feature-reduction criteria

Let $A=\{a_1,a_2,\ldots ,a_r\}$ be the full set of extracted time-series features. In industrial signal analysis, *A* may contain many statistical, temporal, spectral and complexity-based descriptors. Although a large feature set may contain useful information, it can also introduce redundancy, noise sensitivity and unstable neighborhoods. In neighborhood rough-set models, the choice of features directly affects the dissimilarity $\delta _{B,t}$ in (21), the dynamic neighborhoods in (24), and hence the lower, upper and boundary approximations in (25). Therefore, feature reduction is not only a dimensionality reduction step; it changes the rough approximation structure itself.

In early-warning applications, the selected features should satisfy three requirements:

(i) they should preserve the ability to classify windows into decision classes;
(ii) they should preserve the boundary-region behavior needed for uncertainty-based warning;
(iii) they should remain stable across time, rather than being informative only in isolated windows.

The dynamic rough feature-reduction method developed below is designed to satisfy these requirements.

#### Dynamic dependency profile

For each $B\subseteq A$, the dynamic dependency degree

$$\begin{aligned} \gamma _{B,t}^{\rho _t}(d) \end{aligned}$$

defined in (32) measures the proportion of objects in $U_t$ that can be certainly classified by the features in *B*. Instead of using only its average value, it is useful to consider the full dependency profile

68 $$\begin{aligned} \mathbf {\Gamma }_B(d) = \bigl ( \gamma _{B,1}^{\rho _1}(d), \gamma _{B,2}^{\rho _2}(d), \ldots , \gamma _{B,T}^{\rho _T}(d) \bigr )\in [0,1]^T. \end{aligned}$$

The temporal dependency degree in (33) is the arithmetic mean of this profile:

69 $$\begin{aligned} \Gamma _B(d) = \frac{1}{T} \sum _{t=1}^{T}\gamma _{B,t}^{\rho _t}(d). \end{aligned}$$

The full profile in (68) is important because two feature subsets may have the same mean dependency degree but different temporal behavior. For example, one subset may perform uniformly well over all windows, while another may perform well only near the abnormal event. In early-warning systems, such differences should not be ignored.

To measure the loss of decision ability caused by using *B* instead of *A*, define the temporal dependency loss

70 $$\begin{aligned} \mathcal {L}_\textrm{dep}(B) = \Gamma _A(d)-\Gamma _B(d). \end{aligned}$$

By (35), one has

$$\begin{aligned} 0\le \Gamma _B(d)\le 1 \end{aligned}$$

for every $B\subseteq A$. The loss in (70) therefore measures how much average rough decision ability is lost after feature reduction.

#### Pointwise temporal preservation

The average loss in (70) may hide poor behavior at specific time points. Therefore, a stronger pointwise preservation condition is introduced. For $\eta \ge 0$, define

71 $$\begin{aligned} \mathcal {L}_\textrm{pt}(B) = \max _{1\le t\le T} \Big ( \gamma _{A,t}^{\rho _t}(d)-\gamma _{B,t}^{\rho _t}(d) \Big ). \end{aligned}$$

A subset $B\subseteq A$ satisfies pointwise $\eta$-preservation if

72 $$\begin{aligned} \mathcal {L}_\textrm{pt}(B)\le \eta . \end{aligned}$$

Condition (72) is stricter than the average condition in (70). It requires that the reduced feature set preserve the rough decision ability of the full feature set at every time index up to the tolerance $\eta$. This is useful when the objective is to avoid weak performance during short but critical pre-event intervals.

##### Definition 5

Let $\eta \ge 0$. A subset $B\subseteq A$ is called a strong $\eta$-temporal reduct of *A* with respect to *d* if

73 $$\begin{aligned} \max _{1\le t\le T} \Big ( \gamma _{A,t}^{\rho _t}(d)-\gamma _{B,t}^{\rho _t}(d)\Big ) \le \eta \end{aligned}$$

and no proper subset $C\subsetneq B$ satisfies (73).

The strong temporal reduct in Definition 5 strengthens the temporal reduct in Definition 3. The definition is useful when the application requires reliable behavior across all windows, not only on average.

#### Boundary-preserving feature reduction

Since the present paper uses the boundary region for early warning, preserving only the positive region may be insufficient. A feature subset may preserve classification accuracy while distorting the boundary region and therefore weakening the warning mechanism. To address this issue, a boundary-preservation criterion is introduced.

For a target risk class $D_{c,t}$, let $BND_{B,t}(D_{c,t})$ be the dynamic boundary region defined in (55). The normalized boundary size is

74 $$\begin{aligned} b_{B,t}(D_{c,t}) = \frac{|BND_{B,t}(D_{c,t})|}{|U_t|}. \end{aligned}$$

For a set of risk-related classes $\mathcal {C}_\textrm{risk}$, define the total risk-boundary size

75 $$\begin{aligned} b_{B,t}^\textrm{risk} = \sum _{c\in \mathcal {C}_\textrm{risk}} \alpha _c\, b_{B,t}(D_{c,t}), \qquad \alpha _c\ge 0,\quad \sum _{c\in \mathcal {C}_\textrm{risk}}\alpha _c=1. \end{aligned}$$

The boundary-preservation loss of *B* is then defined by

76 $$\begin{aligned} \mathcal {L}_\textrm{bnd}(B) = \frac{1}{T} \sum _{t=1}^{T} \Big | b_{A,t}^\textrm{risk}-b_{B,t}^\textrm{risk} \Big |. \end{aligned}$$

A small value of $\mathcal {L}_\textrm{bnd}(B)$ means that the reduced feature subset preserves the boundary-region behavior of the full feature set. This is especially important because the rough early-warning index in (29) is derived from the gap between lower and upper approximations.

#### Warning-index preservation

The rough early-warning index should also be preserved after feature reduction. Let

$$\begin{aligned} E_{B,t}^\textrm{risk} \end{aligned}$$

be the risk index computed from the feature subset *B*, using either (57) or (58). Define the warning-index preservation loss by

77 $$\begin{aligned} \mathcal {L}_\textrm{warn}(B) = \frac{1}{T} \sum _{t=1}^{T} \Big | E_{A,t}^\textrm{risk}-E_{B,t}^\textrm{risk} \Big |. \end{aligned}$$

The quantity in (77) measures how closely the reduced feature subset reproduces the early-warning trajectory generated by the full feature set. In early-warning applications, this criterion is useful because the final objective is not only to preserve static classification ability, but also to preserve the temporal uncertainty signal.

#### Redundancy control

A feature subset may preserve dependency but still contain redundant variables. To control redundancy, a similarity penalty is introduced. Let

$$\begin{aligned} \textrm{corr}(a_i,a_j) \end{aligned}$$

denote the absolute Pearson correlation between the values of features $a_i$ and $a_j$ over the training windows. Other dependence measures may also be used, such as mutual information or rank-based correlation. The redundancy score of *B* is

78 $$\begin{aligned} \mathcal {R}(B) = {\left\{ \begin{array}{ll} \dfrac{2}{|B|(|B|-1)} \displaystyle \sum _{\begin{array}{c} a_i,a_j\in B\\ i<j \end{array}} |\textrm{corr}(a_i,a_j)|, & |B|\ge 2,\\ 0, & |B|\le 1. \end{array}\right. } \end{aligned}$$

A smaller value of $\mathcal {R}(B)$ indicates less redundancy among the selected features. Redundancy control is common in feature-selection methodology and is consistent with the objective of selecting compact and interpretable reducts^5^.

#### Dynamic rough feature-selection objective

The previous criteria are combined into a single objective function. For $B\subseteq A$, define

79 $$\begin{aligned} \mathcal {J}(B) = \lambda _1\mathcal {L}_\textrm{dep}(B) + \lambda _2\mathcal {L}_\textrm{bnd}(B) + \lambda _3\mathcal {L}_\textrm{warn}(B) + \lambda _4\mathcal {R}(B) + \lambda _5\frac{|B|}{|A|}, \end{aligned}$$

where

$$\begin{aligned} \lambda _i\ge 0,\qquad i=1,2,\ldots ,5, \qquad \sum _{i=1}^{5}\lambda _i=1. \end{aligned}$$

The terms in (79) have the following roles:

(i) $\mathcal {L}_\textrm{dep}(B)$ preserves rough classification ability;
(ii) $\mathcal {L}_\textrm{bnd}(B)$ preserves boundary-region behavior;
(iii) $\mathcal {L}_\textrm{warn}(B)$ preserves the early-warning trajectory;
(iv) $\mathcal {R}(B)$ penalizes redundant features;
(v) |*B*|/|*A*| encourages compactness.

The selected dynamic rough feature subset is

80 $$\begin{aligned} B^*\in \arg \min _{B\subseteq A} \mathcal {J}(B). \end{aligned}$$

Since the exact minimization in (80) is combinatorial, a greedy approximation is used in the algorithmic implementation.

### Algorithm, complexity and interpretation

The dynamic rough feature-reduction procedure consists of two stages. The first stage is forward selection. Starting from the empty set, the method adds the feature that gives the largest improvement in $\mathcal {J}$. The second stage is backward pruning. After a candidate subset is obtained, each selected feature is tested for removal. If removing a feature does not violate the temporal preservation constraint, the feature is removed.

For $B\subseteq A$ and $a\in A\setminus B$, define the forward improvement

81 $$\begin{aligned} G_\textrm{add}(a\mid B) = \mathcal {J}(B)-\mathcal {J}(B\cup \{a\}). \end{aligned}$$

The best feature to add is

82 $$\begin{aligned} a^*\in \arg \max _{a\in A\setminus B} G_\textrm{add}(a\mid B). \end{aligned}$$

A feature is added if $G_\textrm{add}(a^*\mid B)>0$. For pruning, define the removal loss

83 $$\begin{aligned} G_\textrm{rem}(a\mid B) = \mathcal {J}(B\setminus \{a\})-\mathcal {J}(B). \end{aligned}$$

Removing *a* is acceptable if the objective does not increase significantly and the preservation constraint remains satisfied.

The preservation constraint used in pruning is

84 $$\begin{aligned} \mathcal {L}_\textrm{dep}(B)\le \eta _\textrm{dep}, \qquad \mathcal {L}_\textrm{warn}(B)\le \eta _\textrm{warn}, \end{aligned}$$

where $\eta _\textrm{dep}\ge 0$ and $\eta _\textrm{warn}\ge 0$ are validation-selected tolerances. The first condition preserves decision ability, while the second preserves the rough early-warning trajectory.

#### Dynamic feature weights

The dissimilarity in (21) uses time-dependent weights $w_{a,t}$. These weights may be fixed uniformly, learned from validation data, or derived from the contribution of each feature to the dynamic dependency degree. A simple rough-dependency-based weighting scheme is

85 $$\begin{aligned} S_{a,t} = \gamma _{\{a\},t}^{\rho _t}(d), \end{aligned}$$

where $S_{a,t}$ measures the individual rough dependency of feature *a* at time *t*. The normalized weight is

86 $$\begin{aligned} w_{a,t} = \frac{S_{a,t}+\varepsilon }{\sum _{b\in A}(S_{b,t}+\varepsilon )}. \end{aligned}$$

Here, $\varepsilon >0$ is a small numerical constant. The weights in (86) satisfy

$$\begin{aligned} 0<w_{a,t}<1, \qquad \sum _{a\in A}w_{a,t}=1. \end{aligned}$$

If stability is desired, the weights may be smoothed over time:

87 $$\begin{aligned} \bar{w}_{a,t} = \lambda _w w_{a,t} + (1-\lambda _w)\bar{w}_{a,t-1}, \qquad 0<\lambda _w\le 1, \end{aligned}$$

with $\bar{w}_{a,1}=w_{a,1}$. The smoothed weights $\bar{w}_{a,t}$ may then be used in (21). This gives a feature-weighting mechanism that remains consistent with the rough dependency structure.

#### Feature-selection algorithm

The full dynamic rough feature-reduction procedure is summarized in Algorithm 2. The algorithm uses training and validation data only. The test set is not used for selecting $B^*$, tuning the thresholds, estimating normalization constants, or choosing feature weights.

**Algorithm 2:**
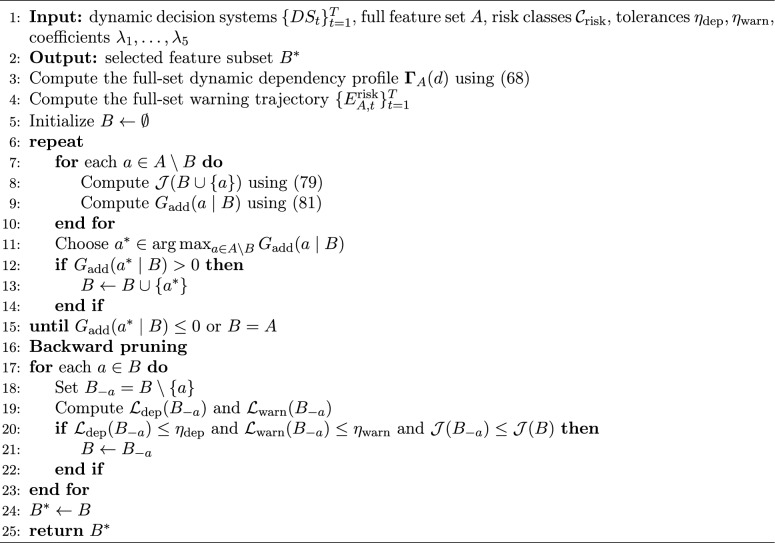
Dynamic rough feature reduction.

#### Complexity analysis

Let $n_t=|U_t|$, $r=|A|$, and *T* be the number of time indices. Computing all pairwise distances in $U_t$ for one feature subset has complexity

$$\begin{aligned} O(n_t^2|B|). \end{aligned}$$

Computing dynamic neighborhoods, lower approximations and upper approximations also requires checking neighborhood membership and decision-class membership. Therefore, the dominant cost for one subset *B* over all time indices is

88 $$\begin{aligned} O\Big ( \sum _{t=1}^{T} n_t^2 |B| \Big ). \end{aligned}$$

In the forward-selection stage, at most *r* features are added. At each step, up to *r* candidate features may be evaluated. Thus, the worst-case number of subset evaluations is $O(r^2)$. The overall worst-case complexity is therefore

89 $$\begin{aligned} O\Big ( r^2\sum _{t=1}^{T} n_t^2 r \Big ) = O\Big ( r^3\sum _{t=1}^{T} n_t^2 \Big ), \end{aligned}$$

where the factor *r* comes from the maximum possible size of a candidate subset.

In practice, the cost can be reduced by precomputing single-feature distance matrices. If

$$\begin{aligned} D_{a,t}(x,y)=\delta _{a,t}(f_t(x,a),f_t(y,a)), \end{aligned}$$

then

$$\begin{aligned} \delta _{B,t}(x,y)=\sum _{a\in B}w_{a,t}D_{a,t}(x,y). \end{aligned}$$

This allows candidate subsets to be evaluated by summing precomputed matrices rather than recomputing distances from raw feature values. Approximate nearest-neighbor search may also be used for very large datasets.

#### Interpretation of selected features

The selected subset $B^*$ has a direct interpretation. A feature $a\in B^*$ is retained because it contributes to at least one of the following:

(i) preserving the dynamic dependency degree;
(ii) preserving the boundary region of risk-related classes;
(iii) preserving the early-warning index trajectory;
(iv) reducing redundancy while maintaining rough decision ability.

Thus, the output of the feature-reduction stage is not merely a list of variables. It also explains which features are needed to maintain the dynamic rough approximation structure. This is important for scientific interpretation. In industrial data, selected features may identify vibration, sensor or degradation descriptors that become informative before failure.

#### Final reduced dynamic rough model

After feature reduction, the complete model in (67) is replaced by the reduced dynamic rough model

90 $$\begin{aligned} \mathfrak {D}_{B^*} = \bigl ( \{DS_t|_{B^*}\}_{t=1}^{T}, B^*, \{\delta _{B^*,t}\}_{t=1}^{T}, \{\rho _{B^*,t}\}_{t=1}^{T}, \{\underline{R}_{B^*,t},\overline{R}_{B^*,t},BND_{B^*,t}\}_{t=1}^{T}, \{E_{B^*,t}^\textrm{risk}\}_{t=1}^{T} \bigr ), \end{aligned}$$

where $DS_t|_{B^*}$ denotes the restriction of the dynamic decision system $DS_t$ to the selected feature subset $B^*$.

The reduced model $\mathfrak {D}_{B^*}$ is the model used in the experimental section. It preserves the rough decision structure of the full feature set as much as possible while improving interpretability, reducing redundancy and lowering computational cost.

## Algorithmic framework

This section presents the complete algorithmic framework used to implement the proposed dynamic rough set learning model. The framework integrates window construction, feature extraction, normalization, dynamic rough approximation, feature reduction, validation-based parameter selection, and final inference. The mathematical objects used below were introduced in the previous sections: the sliding-window representation in (16), the feature map in (17), the dynamic decision system in (19), the dynamic neighborhood relation in (23), the dynamic approximations in (25), the rough early-warning index in (29), and the reduced model in (90).

The purpose of the algorithmic framework is to make the proposed method reproducible. In particular, the training, validation and test stages are separated carefully so that no information from the test set is used for normalization, feature selection, threshold tuning or neighborhood-radius selection. This is essential in industrial time-series experiments, where data leakage may lead to overoptimistic conclusions. The framework follows the rough-set idea of transparent approximation^1^, the neighborhood rough-set treatment of numerical data^7,8^, and the three-way decision interpretation of uncertain cases^2,3^.

### Data preparation and reference-system construction

The input of the framework is a collection of labelled or event-annotated time series

$$\begin{aligned} \mathcal {D}= \{(\mathcal {X}^{(i)},y^{(i)}) : i=1,2,\ldots ,N\}, \end{aligned}$$

where $\mathcal {X}^{(i)}$ is the *i*-th time series and $y^{(i)}$ denotes either a class label, a sequence of labels, or an event time. If event times are available, warning labels are generated using (48). If only ordinary class labels are available, binary or multiclass labels are used as in (49).

The final output is not only a predicted class. The framework returns

(i) the selected feature subset $B^*$;
(ii) the reduced dynamic rough model $\mathfrak D_{B^*}$ in (90);
(iii) the rough decision of each test window;
(iv) the boundary-region trajectory of the risk-related classes;
(v) the rough early-warning trajectory;
(vi) the final warning times and performance measures.

#### Data partitioning

The dataset is divided into three disjoint parts:

91 $$\begin{aligned} \mathcal {D} = \mathcal {D}_\textrm{train} \cup \mathcal {D}_\textrm{val} \cup \mathcal {D}_\textrm{test}, \qquad \mathcal {D}_\textrm{train}\cap \mathcal {D}_\textrm{val} = \mathcal {D}_\textrm{train}\cap \mathcal {D}_\textrm{test} = \mathcal {D}_\textrm{val}\cap \mathcal {D}_\textrm{test} = \emptyset . \end{aligned}$$

The training set is used to estimate normalization constants, construct reference neighborhoods, compute feature-reduction quantities and fit any auxiliary baseline classifier. The validation set is used to choose hyperparameters such as the window length, stride, neighborhood quantile, smoothing coefficients and warning thresholds. The test set is used only once for the final evaluation.

For time-series data, the split should be performed at the subject, machine or trajectory level whenever possible. For example, in industrial degradation data, windows from the same engine unit or machine run should not be distributed across training, validation and test splits. This prevents window-level leakage and gives a more realistic estimate of generalization.

#### Preprocessing

Each time series is first checked for missing values, corrupted samples and extreme artifacts. Missing values may be handled by a training-only imputation rule. Let $x_{\ell ,q}^{(i)}$ be the value of the *q*-th channel at time $\ell$ in the *i*-th series. If a value is missing, it is replaced by

92 $$\begin{aligned} \widetilde{x}_{\ell ,q}^{(i)} = {\left\{ \begin{array}{ll} x_{\ell ,q}^{(i)}, & x_{\ell ,q}^{(i)}\ \text {is observed}, \\ \textrm{Med}_{q}^\textrm{train}, & x_{\ell ,q}^{(i)}\ \text {is missing}, \end{array}\right. } \end{aligned}$$

where $\textrm{Med}_{q}^\textrm{train}$ is the median of the *q*-th channel estimated from the training set only. Other imputation methods may be used, but their parameters must also be estimated from the training set only.

After imputation, windows are constructed using (16). Feature vectors are extracted using (17). The feature values are normalized by the training-only min–max transformation in (46). The same normalization constants are then applied to validation and test windows.

#### Construction of dynamic reference systems

For each time index *t*, the training windows define a labelled reference universe

93 $$\begin{aligned} \mathcal {R}_t = \{u_{i,t}: \mathcal {X}^{(i)}\in \mathcal {D}_\textrm{train},\ W_{i,t}\ \text {is defined}\}. \end{aligned}$$

The corresponding labelled reference decision system is

94 $$\begin{aligned} RS_t= (\mathcal {R}_t,A\cup \{d\},V_t,f_t). \end{aligned}$$

The reference system $RS_t$ is used to compute the dynamic neighborhoods, lower approximations, upper approximations, boundary regions and dependency degrees during training. It is also used during test-time inference so that the labels of test windows are never used to define their neighborhoods.

For a validation or test window *x*, its reference neighborhood is defined by

95 $$\begin{aligned} \mathcal {N}_{B,t}^{\rho _t}(x;\mathcal {R}_t) = \{y\in \mathcal {R}_t:\delta _{B,t}(x,y)\le \rho _t\}. \end{aligned}$$

If $\mathcal {N}_{B,t}^{\rho _t}(x;\mathcal {R}_t)=\emptyset$, a $k_0$-nearest-neighbor fallback is used:

96 $$\begin{aligned} \mathcal {N}_{B,t}^{\rho _t}(x;\mathcal {R}_t) = \mathcal {N}_{B,t}^{(k_0)}(x;\mathcal {R}_t), \end{aligned}$$

where $\mathcal {N}_{B,t}^{(k_0)}(x;\mathcal {R}_t)$ denotes the set of the $k_0$ nearest reference windows to *x* with respect to $\delta _{B,t}$. This fallback prevents division by zero in local support calculations.

### Training, validation and inference

The training stage constructs the full dynamic rough model using the full feature set *A*. For every time *t*, pairwise distances are computed on the reference universe $\mathcal {R}_t$ using (21). The adaptive radius is selected by the quantile rule in (52), optionally smoothed by (53). Dynamic neighborhoods are then constructed using (24).

For each decision class $D_{c,t}$, the lower and upper approximations are computed by (25), and the boundary region is computed by (26). The rough early-warning index is then obtained from (29). If the problem is binary, the target warning index is $E_t^+$ from (56). If the problem has warning and abnormal classes, the risk index is computed by (57) or (58).

The training stage also computes the quantities required for dynamic rough feature reduction. In particular, the dynamic dependency profile in (68), the temporal dependency loss in (70), the boundary-preservation loss in (76), and the warning-index preservation loss in (77) are computed using the training reference systems. Algorithm 2 then returns the selected subset $B^*$.

#### Validation stage and parameter selection

Let

97 $$\begin{aligned} \Theta = (h,s,q_\rho ,\lambda _\rho ,L,\theta _E,\theta _\Delta ,\theta _G,k_0,\eta _\textrm{dep},\eta _\textrm{warn}) \end{aligned}$$

denote the algorithmic parameter vector. These parameters are chosen using the validation set only. For each candidate value of $\Theta$, the model is trained on $\mathcal {D}_\textrm{train}$, feature reduction is performed on the training reference systems, and the resulting model is evaluated on $\mathcal {D}_\textrm{val}$.

The validation objective should reflect both classification and early-warning quality. A convenient validation loss is

98 $$\begin{aligned} \mathcal {J}_\textrm{val}(\Theta ) = \beta _1(1-\textrm{F1}_\textrm{risk}) + \beta _2\textrm{FAR} + \beta _3\textrm{MDR} + \beta _4\textrm{Delay} + \beta _5\textrm{Size}, \end{aligned}$$

where $\textrm{F1}_\textrm{risk}$ is the F1-score for the risk-related class, $\textrm{FAR}$ is the false-alarm rate, $\textrm{MDR}$ is the missed-detection rate, $\textrm{Delay}$ is the average detection delay, and $\textrm{Size}=|B^*|/|A|$ is the normalized selected feature-set size. The coefficients satisfy

$$\begin{aligned} \beta _i\ge 0,\qquad i=1,2,\ldots ,5, \qquad \sum _{i=1}^{5}\beta _i=1. \end{aligned}$$

The selected parameter vector is

99 $$\begin{aligned} \Theta ^*\in \arg \min _{\Theta \in \mathcal {P}} \mathcal {J}_\textrm{val}(\Theta ), \end{aligned}$$

where $\mathcal {P}$ is the validation search space.

#### Early-warning performance measures

Suppose that the *i*-th time series has an event time $\tau _i$. Let

$$\begin{aligned} \mathcal {W}_i = \{t: W_{i,t}\ \text {is defined}\} \end{aligned}$$

be the set of its window indices. The first warning time is

100 $$\begin{aligned} \widehat{\tau }_i = \min \{e_{i,t}:t\in \mathcal {W}_i,\ \textrm{Warning}_{i,t}=1\}, \end{aligned}$$

provided that at least one warning is generated. If no warning is generated, $\widehat{\tau }_i$ is left undefined and the event is counted as missed.

The lead time is

101 $$\begin{aligned} \textrm{LT}_i = \tau _i-\widehat{\tau }_i, \end{aligned}$$

whenever $\widehat{\tau }_i<\tau _i$. A positive lead time means that the warning was generated before the event. The detection delay is

102 $$\begin{aligned} \textrm{DD}_i = \max \{0,\widehat{\tau }_i-\tau _i\}. \end{aligned}$$

The missed-detection rate is

103 $$\begin{aligned} \textrm{MDR} = \frac{\#\{i:\widehat{\tau }_i\ \text {is undefined}\}}{\#\{i:\tau _i\ \text {is available}\}}. \end{aligned}$$

The false-alarm rate may be computed as

104 $$\begin{aligned} \textrm{FAR} = \frac{\#\{(i,t):\textrm{Warning}_{i,t}=1,\ e_{i,t}<\tau _i-\Delta \}}{\#\{(i,t):e_{i,t}<\tau _i-\Delta \}}, \end{aligned}$$

where $\Delta$ is the warning horizon used in (48). These measures evaluate whether the proposed rough uncertainty trajectory gives useful warning before the abnormal event.

#### Test-time inference

During test-time inference, the label of the test window is unknown and must not be used in the construction of its neighborhood. Therefore, the test window is compared only with the labelled training reference universe $\mathcal {R}_t$. For a test window *x*, compute its reference neighborhood by (95). Let $D_{c,t}^{\mathcal {R}}\subseteq \mathcal {R}_t$ denote the reference objects belonging to class *c*. The local rough support of class *c* is

105 $$\begin{aligned} \pi _{c,t}^{\mathcal {R}}(x) = \frac{|\mathcal {N}_{B^*,t}^{\rho _t}(x;\mathcal {R}_t)\cap D_{c,t}^{\mathcal {R}}|}{|\mathcal {N}_{B^*,t}^{\rho _t}(x;\mathcal {R}_t)|}. \end{aligned}$$

The predicted class is

106 $$\begin{aligned} \widehat{d}_t(x) = \arg \max _c \pi _{c,t}^{\mathcal {R}}(x). \end{aligned}$$

The local uncertainty score is

107 $$\begin{aligned} U_t^\textrm{loc}(x) = 1-\max _c \pi _{c,t}^{\mathcal {R}}(x). \end{aligned}$$

A high value of $U_t^\textrm{loc}(x)$ indicates that the reference neighborhood of *x* is mixed across classes. The confidence gap is

108 $$\begin{aligned} G_t^{\mathcal {R}}(x) = \pi _{\widehat{d}_t(x),t}^{\mathcal {R}}(x) - \max _{c\ne \widehat{d}_t(x)} \pi _{c,t}^{\mathcal {R}}(x). \end{aligned}$$

The final rough decision for the test window is

109 $$\begin{aligned} \textrm{Decision}_t(x) = {\left\{ \begin{array}{ll} \textrm{accept}\ \widehat{d}_t(x), & G_t^{\mathcal {R}}(x)\ge \theta _G,\\ \textrm{defer}, & G_t^{\mathcal {R}}(x)<\theta _G. \end{array}\right. } \end{aligned}$$

The test-time warning output combines the local uncertainty score with the learned warning thresholds:

110 $$\begin{aligned} \textrm{Warning}_t(x) = {\left\{ \begin{array}{ll} 1, & U_t^\textrm{loc}(x)\ge \theta _E, \\ 1, & G_t^{\mathcal {R}}(x)<\theta _G\ \text {and}\ \widehat{d}_t(x)\in \mathcal {C}_\textrm{risk}, \\ 0, & \text {otherwise}. \end{array}\right. } \end{aligned}$$

This rule makes the inference stage practically usable, because it does not require the unknown test label. In retrospective evaluation, once the test labels are revealed, the predicted classes and warning times can be compared with the ground truth.

**Fig. 1:**
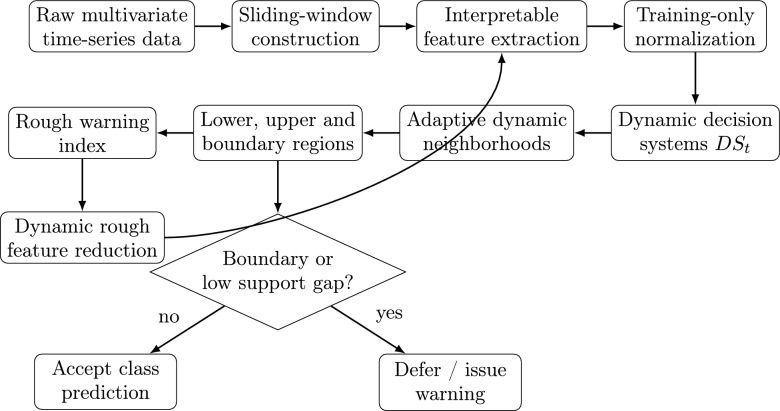
Flowchart of the proposed dynamic rough set early-warning methodology.

Figure 1 summarizes the complete methodology. The flowchart also clarifies where the main proposed rough-set components enter the pipeline: the dynamic neighborhood construction, lower–upper approximation computation, rough early-warning index and dynamic feature reduction.

### Complete procedure, robustness and reproducibility

The complete training, validation and testing procedure is summarized in Algorithm 3.

**Algorithm 3:**
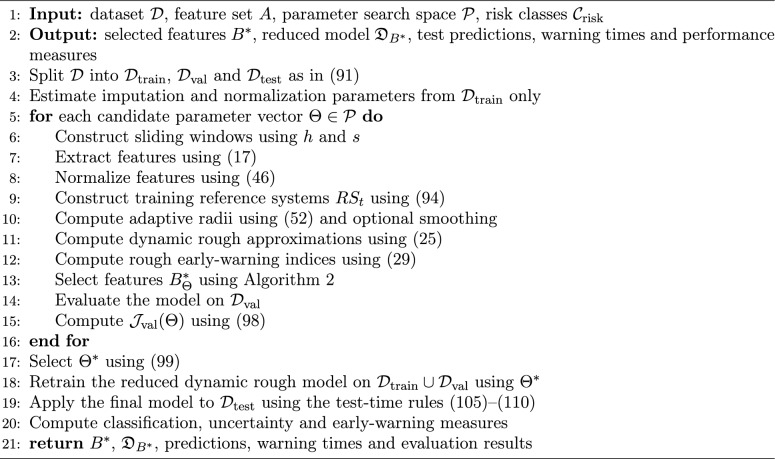
Complete dynamic rough set learning framework.

#### Complexity of the complete framework

Let $n_t=|\mathcal {R}_t|$, $r=|A|$, $b=|B^*|$, and *T* be the number of time indices. For a fixed feature subset *B*, the dominant training cost is the computation of pairwise distances on each reference universe:

111 $$\begin{aligned} O\Big (\sum _{t=1}^{T} n_t^2 |B|\Big ). \end{aligned}$$

The feature-reduction stage has worst-case complexity of the form described in (89). After the selected subset $B^*$ has been fixed, the cost of classifying a single test window at time *t* is

112 $$\begin{aligned} O(n_t b), \end{aligned}$$

because the test window is compared with the $n_t$ labelled reference windows using *b* selected features. Thus, the reduced feature set improves both interpretability and test-time efficiency.

If the reference sets are large, approximate nearest-neighbor search, clustering or prototype selection can be used to reduce the inference cost. These acceleration strategies do not change the mathematical definition of the proposed model, but they may be useful for large industrial sensor networks and other sequential monitoring systems

#### Robustness evaluation protocol

To evaluate robustness, the final model should be tested under controlled perturbations. Let $x_{\ell ,q}^{(i)}$ be an observed signal value. A noisy version may be generated by

113 $$\begin{aligned} x_{\ell ,q}^{(i,\sigma )} = x_{\ell ,q}^{(i)} + \sigma \,\xi _{\ell ,q}^{(i)}, \qquad \xi _{\ell ,q}^{(i)}\sim N(0,1), \end{aligned}$$

where $\sigma \ge 0$ controls the noise level. Missingness may be simulated by a binary mask $M_{\ell ,q}^{(i)}\in \{0,1\}$, where

114 $$\begin{aligned} x_{\ell ,q}^{(i,\textrm{miss})} = {\left\{ \begin{array}{ll} x_{\ell ,q}^{(i)}, & M_{\ell ,q}^{(i)}=1, \\ \textrm{missing}, & M_{\ell ,q}^{(i)}=0. \end{array}\right. } \end{aligned}$$

The same imputation rule in (92) is then applied. Class imbalance may be evaluated by subsampling one class or by reporting class-balanced metrics such as macro-F1 and balanced accuracy.

The robustness evaluation reports how classification performance, deferment rate, false-alarm rate, missed-detection rate and average lead time change as noise, missingness or imbalance increases. This is important because real industrial signals are often noisy, incomplete and nonstationary.

#### Reproducibility requirements

For reproducibility, the following quantities should be reported in the experimental section:

(i) dataset splits and whether the split is unit-level, machine-level or window-level;
(ii) window length *h* and stride *s*;
(iii) the full feature list *A* and the selected subset $B^*$;
(iv) normalization and imputation rules;
(v) neighborhood dissimilarity $$\delta _{B,t}$$;
(vi) radius-selection method and value of $q_\rho$;
(vii) smoothing parameters $\lambda _\rho$ and $\lambda _w$, if used;
(viii) thresholds $\theta _E$, $\theta _\Delta$ and $\theta _G$;
(ix) warning horizon $\Delta$;
(x) evaluation metrics and baseline methods.

Reporting these details is necessary because the rough regions, the warning index and the final warning times depend directly on the selected windows, features, radii and thresholds.

#### Summary

The algorithmic framework converts the proposed dynamic rough set model into a complete reproducible pipeline. The training stage constructs labelled reference approximation spaces. The feature-reduction stage selects a compact subset of informative features. The validation stage selects all thresholds and hyperparameters without using the test set. The inference stage classifies each new window by comparing it with labelled reference windows and produces a warning when local rough uncertainty is high or when the risk-related decision is insufficiently confident. Hence, the algorithm produces both predictive outputs and interpretable uncertainty outputs, which is the central purpose of the proposed model.

## Experimental study

This section evaluates the proposed dynamic rough set framework on an industrial degradation benchmark. To improve readability, the experiment is described according to the main decision steps of the proposed algorithm: dataset and labelling, window construction and preprocessing, model selection, baseline comparison and evaluation.

### Step 1: dataset and decision labels

The framework was evaluated on the NASA C-MAPSS FD001 turbofan degradation dataset^22,23^. Each engine trajectory contains operational settings and sensor measurements recorded over successive cycles. In the training trajectories, each engine evolves from normal operation to failure. This makes the dataset suitable for testing early-warning behavior, because windows near failure are expected to be more difficult to distinguish from healthy windows than fully critical windows.

FD001 was selected for three reasons. First, it is a widely used public benchmark for turbofan run-to-failure degradation, which makes the experiment reproducible and comparable. Second, it provides multivariate sequential data with clear temporal ordering and known end-of-life points, which are necessary for constructing window-level warning labels from remaining useful life. Third, FD001 is the least complex C-MAPSS subset because it has one operating condition and one fault mode. This is an appropriate first benchmark for isolating the behavior of the proposed dynamic rough approximation mechanism before extending the framework to more complex multi-condition subsets such as FD002, FD003 and FD004.

Let $L_i$ denote the last observed cycle of the *i*-th training engine and let $e_{i,t}$ be the end cycle of window $W_{i,t}$. The remaining useful life associated with the window is

115 $$\begin{aligned} \textrm{RUL}_{i,t}=L_i-e_{i,t}. \end{aligned}$$

Using the warning horizon $\Delta =30$, the three-class window label is defined by

116 $$\begin{aligned} d(u_{i,t})= {\left\{ \begin{array}{ll} 0, & \textrm{RUL}_{i,t}>2\Delta , \\ 1, & \Delta <\textrm{RUL}_{i,t}\le 2\Delta , \\ 2, & 0\le \textrm{RUL}_{i,t}\le \Delta . \end{array}\right. } \end{aligned}$$

Thus, class 0 denotes healthy operation, class 1 denotes a warning state, and class 2 denotes a critical near-failure state. The warning class is expected to be the most difficult class because it represents the transition between healthy and critical operation.

### Step 2: Window construction, features and preprocessing

The cycle-level data were converted into sliding windows using a window length of $h=30$ cycles and stride $s=5$ cycles. The value $h=30$ was chosen because it matches the warning horizon $\Delta =30$ and therefore gives each window enough recent degradation history to represent one operational warning interval. The stride $s=5$ was used to obtain frequent warning updates without creating a fully redundant one-cycle sliding dataset. Thus, the pair $(h,s)=(30,5)$ balances temporal context, alarm timeliness and computational cost. Each window received the label of its final cycle.

For each window, statistical and temporal descriptors were computed from the operational settings and sensor values. The extracted features included mean, standard deviation, minimum, maximum, root mean square, peak-to-peak amplitude and least-squares slope. Constant features were removed. This produced 3586 windows and 118 nonconstant features. Table 1 summarizes the feature extraction process.

**Table 1:** Summary of the window-level feature extraction process.

Step	Input	Operation	Output
Windowing	Engine cycles and sensor channels	$h=30$ cycles, $s=5$ cycles	Overlapping degradation windows
Window labelling	End cycle $e_{i,t}$ and RUL	Three-class rule with $\Delta =30$	Healthy, warning or critical label
Feature extraction	Operational settings and sensors	Mean, std., min, max, RMS, peak-to-peak, slope	Candidate window descriptors
Filtering	Candidate descriptors	Removal of constant features	118 nonconstant features
Normalization	Training windows only	Min–max scaling	Leakage-controlled normalized features

The resulting window-level class distribution was 2363 healthy windows, 600 warning windows and 623 critical windows. The data were split by engine unit, not by individual windows, in order to avoid placing windows from the same engine trajectory in both training and test sets. This yielded 70 training engines, 15 validation engines and 15 test engines. The corresponding test set contained 532 windows.

All normalization parameters were estimated from the training set only. Validation and test windows were transformed using the training minima and maxima. This procedure avoids leakage from validation or test data into the training process. In the missingness robustness experiment, missing values were imputed using training medians.

### Step 3: model selection and comparative models

The dynamic rough set model was tuned on the validation set. The validation grid included the neighborhood-radius quantile $q_\rho$, the support-gap threshold $\theta _G$, and the local uncertainty threshold $\theta _E$. The radius quantile controls neighborhood size and therefore directly affects the lower and upper approximations. Small values of $q_\rho$ produce local neighborhoods and fewer boundary windows; larger values increase the number of possible neighbors, which can enlarge the boundary region and raise deferment. The support-gap threshold $\theta _G$ controls how separated the largest rough support must be from the second largest support before a class is accepted. The local uncertainty threshold $\theta _E$ controls when boundary expansion is high enough to be reported as a warning. These values were not tuned on the test set.

The best validation setting was

$$\begin{aligned} q_\rho =0.05,\qquad \theta _G=0.05,\qquad \theta _E=0.30. \end{aligned}$$

This configuration was then used for the final test evaluation. The selected value $q_\rho =0.05$ should be interpreted as a relatively local neighborhood radius, suitable for preserving fine degradation transitions in FD001. The thresholds $\theta _G=0.05$ and $\theta _E=0.30$ reflect a cautious but not overly conservative warning policy: they allow deferment for low-support-gap windows while avoiding an excessive warning rate.

The proposed dynamic rough set model was compared with four standard machine-learning baselines: Logistic Regression, Support Vector Machine, Random Forest and XGBoost^24–27^. These baselines were trained on the same normalized window-level features and evaluated on the same test split. The purpose of this comparison is not to claim that the proposed model is always the strongest pure classifier, but to assess the trade-off between predictive performance and explicit uncertainty-aware outputs.

Recent deep-learning early-warning and forecasting models, such as stacked bidirectional GRU, one-dimensional convolutional networks, LSTM-type recurrent networks and transformer-style time-series models, are important reference points for sequential data^14,18^. In the present experiment, the numerical comparison is restricted to feature-based baselines because the proposed rough-set method itself operates on interpretable window descriptors. A qualitative comparison with deep sequential methods is nevertheless included in the discussion, and a full deep-baseline study is identified as a future empirical extension.

### Step 4: evaluation protocol

The main classification metrics were accuracy, balanced accuracy, Macro-F1 and the F1-score of the warning class. Balanced accuracy and Macro-F1 are important because the warning and critical classes are smaller than the healthy class. The warning-class F1-score is reported separately because early warning is practically useful only if the transitional class is not ignored by the model. The proposed rough model was also evaluated using accepted-window accuracy, deferment rate, warning rate, local uncertainty and support gap. Robustness was assessed under Gaussian feature noise with $\sigma \in \{0.01,0.03,0.05,0.10\}$ and simulated missingness rates $r_\textrm{miss}\in \{0.05,0.10,0.20,0.30\}$, with missing values imputed using training medians. No random oversampling was applied; instead, the imbalance effect was monitored through class-sensitive metrics and validation-based threshold selection.

## Results

This section reports the empirical results obtained on the held-out C-MAPSS FD001 test set after validation-based parameter selection.

### Overall classification and uncertainty-aware performance

Table 2 summarizes the final test performance. In terms of Macro-F1, the strongest pure classification baseline was Random Forest, which achieved an accuracy of 0.9041, balanced accuracy of 0.8779, Macro-F1 score of 0.8715, and warning-class F1-score of 0.7385. XGBoost also performed strongly, with an accuracy of 0.9098 and Macro-F1 score of 0.8611.

The full dynamic rough set model achieved an accuracy of 0.8647, balanced accuracy of 0.7570, Macro-F1 score of 0.7895, and warning-class F1-score of 0.5610. Thus, the full dynamic rough set (DRS) model did not exceed the strongest black-box baseline in raw classification performance. Its added value is that it provides rough-set uncertainty outputs, including local support gaps, local uncertainty, deferred decisions and warning rates.

**Table 2:** Final experimental summary on the C-MAPSS FD001 test set. The proposed DRS models provide additional uncertainty outputs through deferment and warning rates, while the machine-learning baselines provide only direct class predictions.

Method	Features	Reduction	Accuracy	Balanced acc.	Macro-F1	Warning F1	Deferment
Random Forest	118	0.0000	0.9041	0.8779	0.8715	0.7385	–
XGBoost	118	0.0000	0.9098	0.8505	0.8611	0.7209	–
Logistic Regression	118	0.0000	0.8722	0.8645	0.8371	0.6852	–
Support Vector Machine	118	0.0000	0.8759	0.8497	0.8343	0.6667	–
Full DRS	118	0.0000	0.8647	0.7570	0.7895	0.5610	0.0207
Reduced DRS	40	0.6610	0.8459	0.7281	0.7564	0.5067	0.0301

Table 3 places the proposed model in relation to recent deep sequential early-warning approaches. The comparison is conceptual rather than a new numerical deep-learning benchmark. Its purpose is to clarify that the proposed method is designed to add rough uncertainty and deferment information to early-warning decisions, whereas deep recurrent or convolutional models are usually optimized mainly for predictive accuracy or forecasting loss.

**Table 3:** Conceptual comparison with representative deep sequential early-warning and forecasting methods.

Model family	Main strength	Typical limitation for deployment	Relation to the proposed DRS model
1D-CNN / CNN–GRU	Automatic local pattern extraction	Latent features may be hard to inspect	DRS uses explicit sensor statistics
LSTM / BiGRU	Long-range sequential dependence	Requires larger training effort and tuning	DRS uses transparent neighborhoods
Stacked BiGRU with smoothing/decomposition	Robust sequential forecasting under noise	More complex architecture and preprocessing	DRS gives boundary-region uncertainty
Transformer-style time-series models	Flexible sequence representation	Data- and computation-demanding	DRS is lighter and explanation-oriented

The comparative results show that the proposed dynamic rough set model is not primarily advantageous when the only objective is maximum raw classification accuracy. Tree-based ensemble models are stronger for that purpose on FD001. The strength of the proposed method appears in problems where a decision maker needs to distinguish between confident and uncertain predictions. Therefore, the framework is especially suitable for: (i) early-warning tasks with transitional classes, (ii) safety-critical monitoring where ambiguous decisions should be deferred, (iii) industrial datasets where interpretable sensor-derived features are preferred to latent representations, and (iv) maintenance decision support where support gaps, boundary membership and warning rates can guide inspection priorities.

**Fig. 2:**
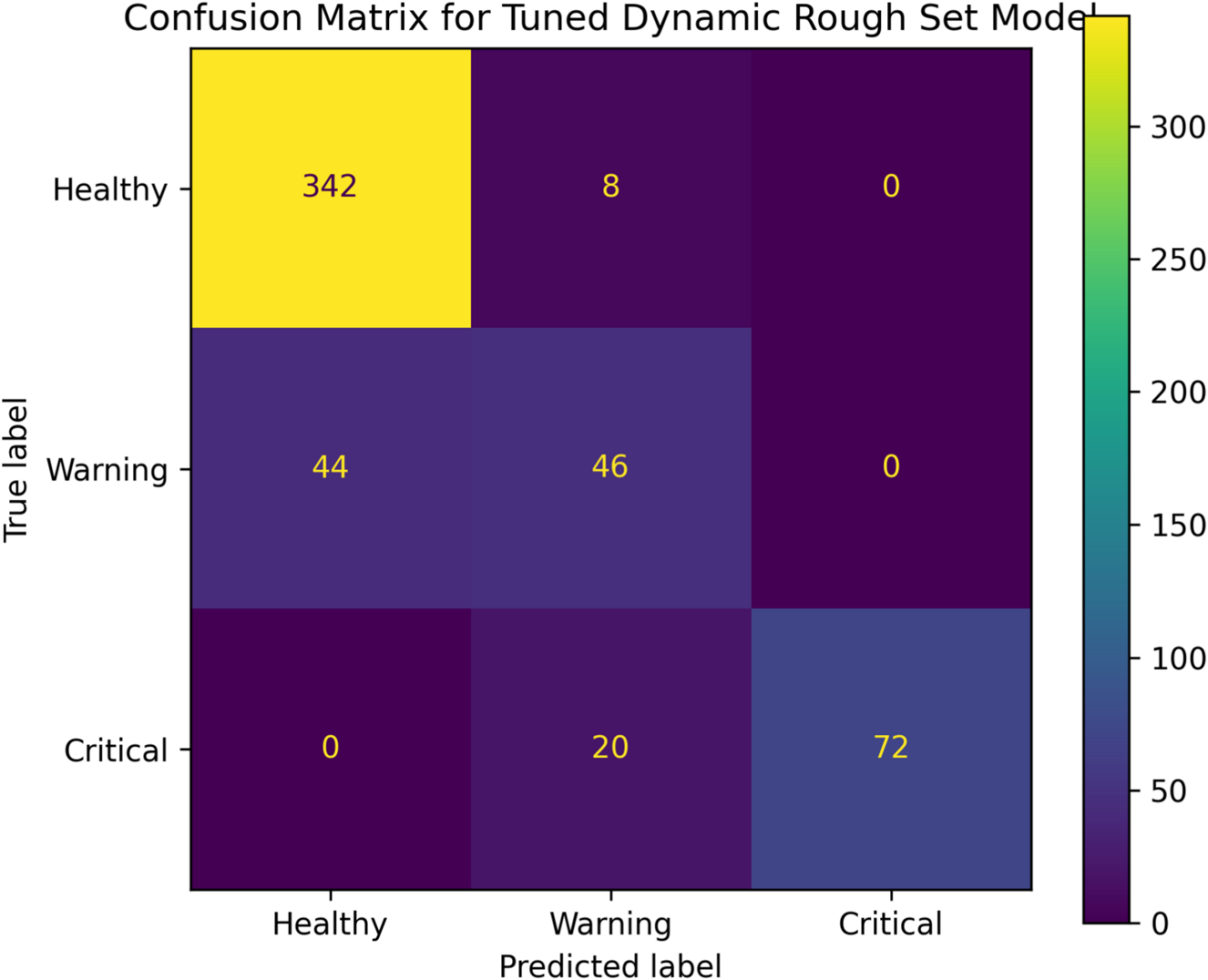
Confusion matrix of the tuned full dynamic rough set model on the C-MAPSS FD001 test set.

Figure 2 shows the confusion matrix of the tuned full dynamic rough set model. The model performs well on the healthy and critical classes, while the warning class remains the most difficult class. This behavior is expected because the warning class represents an intermediate degradation state.

### Uncertainty and deferment analysis

The tuned full DRS model accepted 521 of the 532 test windows and deferred 11 windows, corresponding to a deferment rate of 0.0207. The warning rate was 0.1654. The accepted-window accuracy was 0.8752, which is higher than the overall accuracy of 0.8647. This indicates that the rough support-gap mechanism separated a small subset of less certain windows (Table 4).

**Table 4:** Uncertainty summary of the tuned dynamic rough set model on the C-MAPSS FD001 test set.

Quantity	Value
Number of test windows	532
Accepted windows	521
Deferred windows	11
Deferment rate	0.0207
Warning windows	88
Warning rate	0.1654
Mean local uncertainty	0.1229
Mean support gap	0.7639

**Fig. 3:**
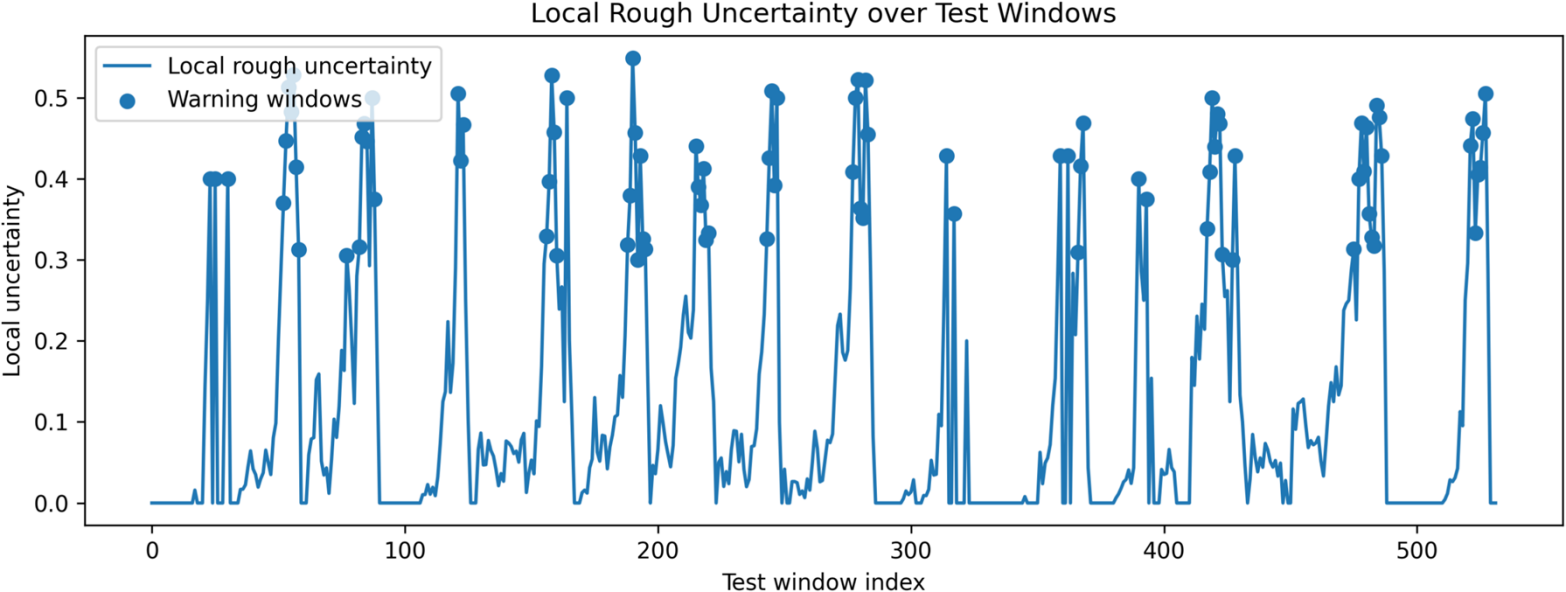
Local rough uncertainty over the C-MAPSS FD001 test windows. Marked points indicate windows for which the model generated a warning.

**Fig. 4:**
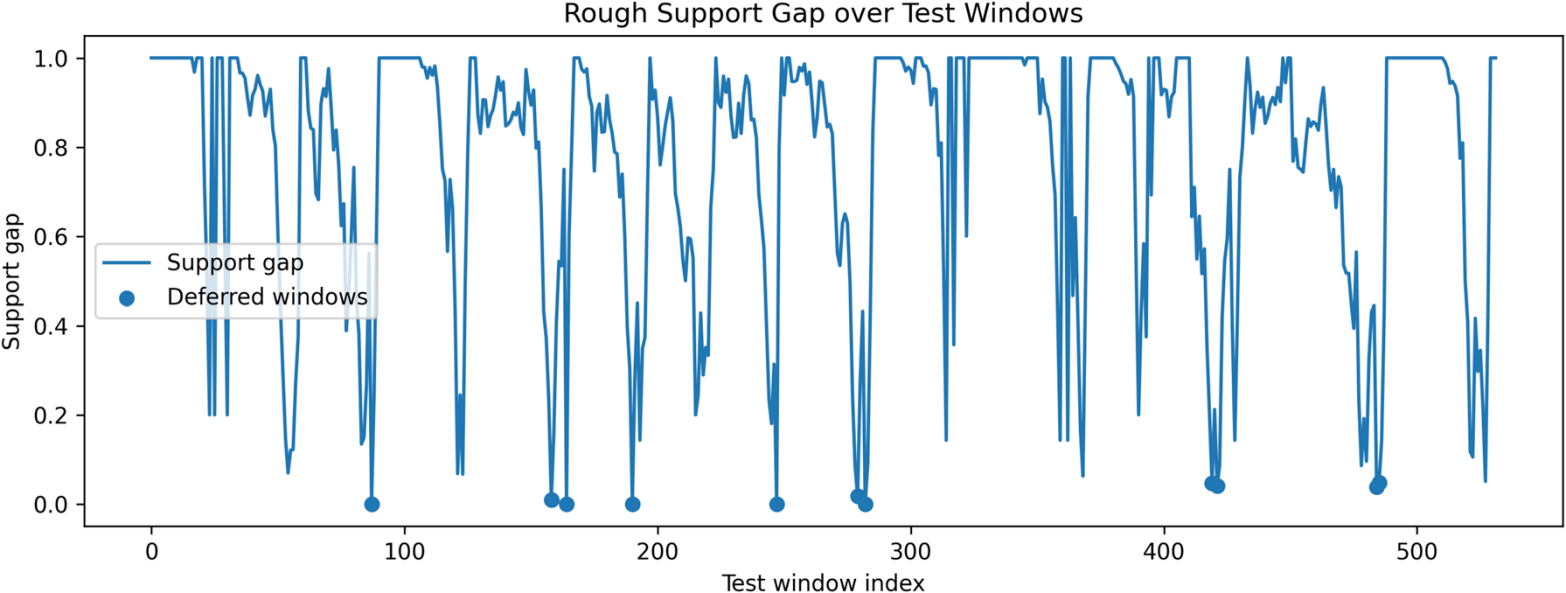
Rough support-gap trajectory over the C-MAPSS FD001 test windows. Smaller support gaps correspond to less decisive local neighborhood evidence.

Figures 3 and 4 show the local uncertainty and support-gap trajectories over the test windows. These figures provide visual evidence that the proposed model does not only return class labels, but also produces window-level uncertainty indicators.

### Dynamic rough feature reduction

Dynamic rough feature reduction selected 40 features from the original 118 features, corresponding to a reduction ratio of 0.6610. The reduced DRS model achieved an accuracy of 0.8459, balanced accuracy of 0.7281, Macro-F1 score of 0.7564, and warning-class F1-score of 0.5067. Therefore, feature reduction produced a moderate decrease in performance, but retained a substantial part of the decision behavior while improving compactness and interpretability.

**Fig. 5:**
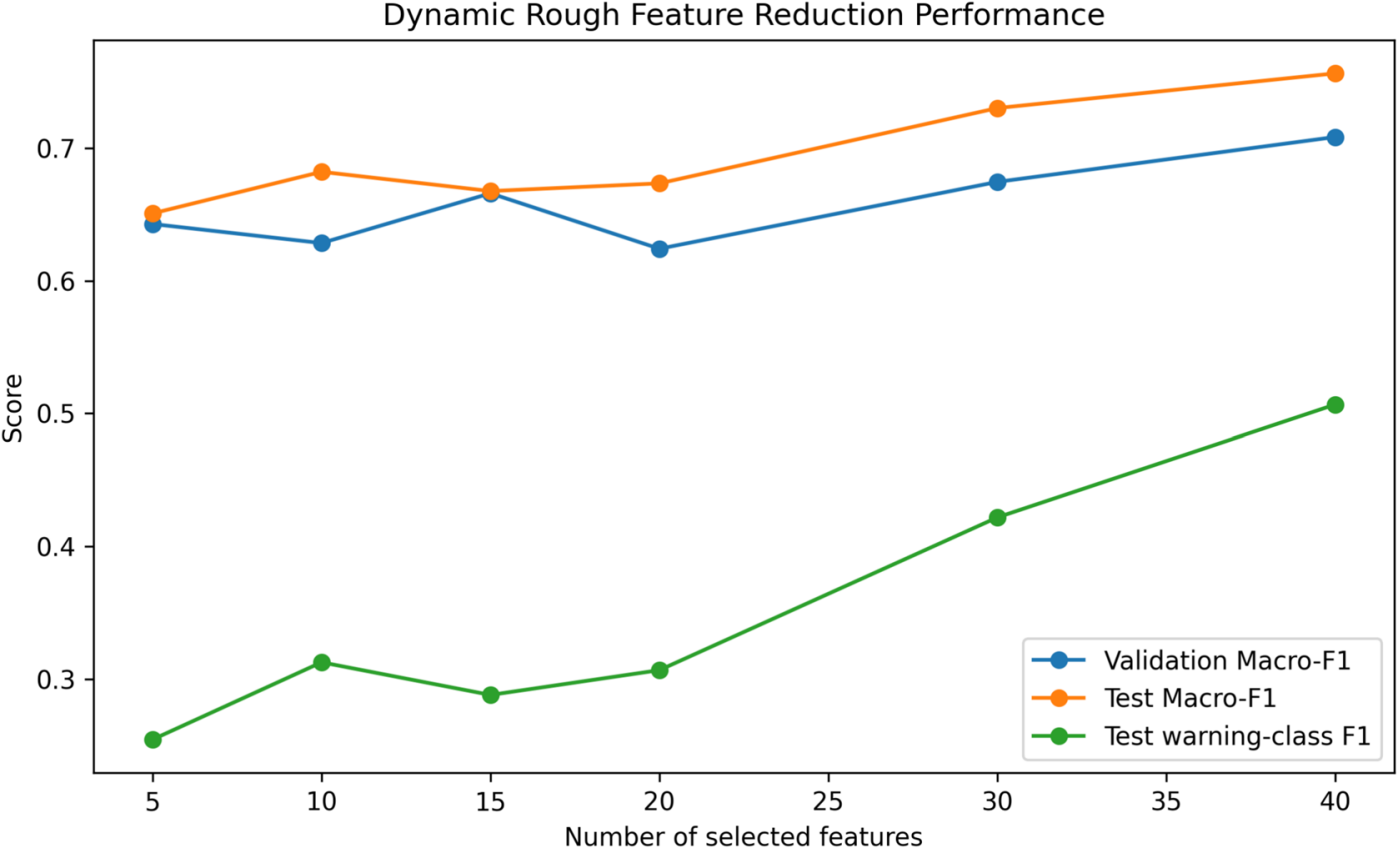
Validation and test performance as a function of the number of selected rough-dependency features .

**Fig. 6:**
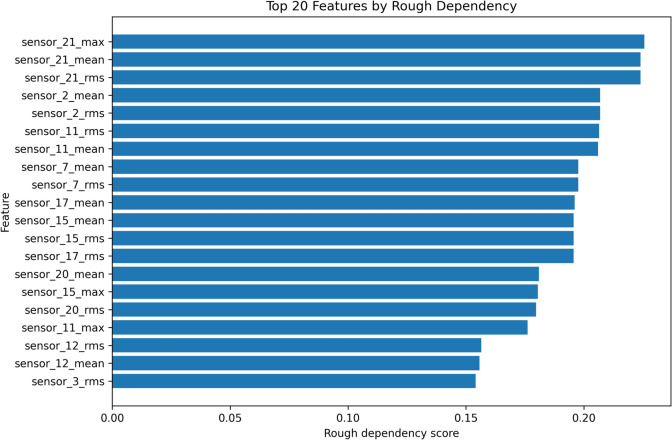
Top 20 features ranked by rough dependency on the C-MAPSS FD001 training set.

Table 5 lists the top 15 features ranked by one-feature rough dependency. The ranking is dominated by interpretable sensor statistics, especially descriptors derived from sensors 21, 2, 11, 7, 17, 15 and 20 (Fig. 5, 6).

**Table 5:** Top 15 features ranked by one-feature rough dependency on the C-MAPSS FD001 training set.

Rank	Feature	Dependency	Positive region size
1	sensor_21_max	0.2256	565
2	sensor_21_mean	0.2240	561
3	sensor_21_rms	0.2240	561
4	sensor_2_mean	0.2069	518
5	sensor_2_rms	0.2069	518
6	sensor_11_rms	0.2065	517
7	sensor_11_mean	0.2061	516
8	sensor_7_mean	0.1977	495
9	sensor_7_rms	0.1977	495
10	sensor_17_mean	0.1961	491
11	sensor_15_mean	0.1957	490
12	sensor_15_rms	0.1957	490
13	sensor_17_rms	0.1957	490
14	sensor_20_mean	0.1809	453
15	sensor_15_max	0.1805	452

### Robustness analysis

Table 6 reports robustness under feature noise and missingness. The tuned DRS model was stable under Gaussian feature noise. At $\sigma =0.10$, the model retained a Macro-F1 score of 0.7888 and a warning-class F1-score of 0.5838, which are close to the clean-test values.

**Fig. 7:**
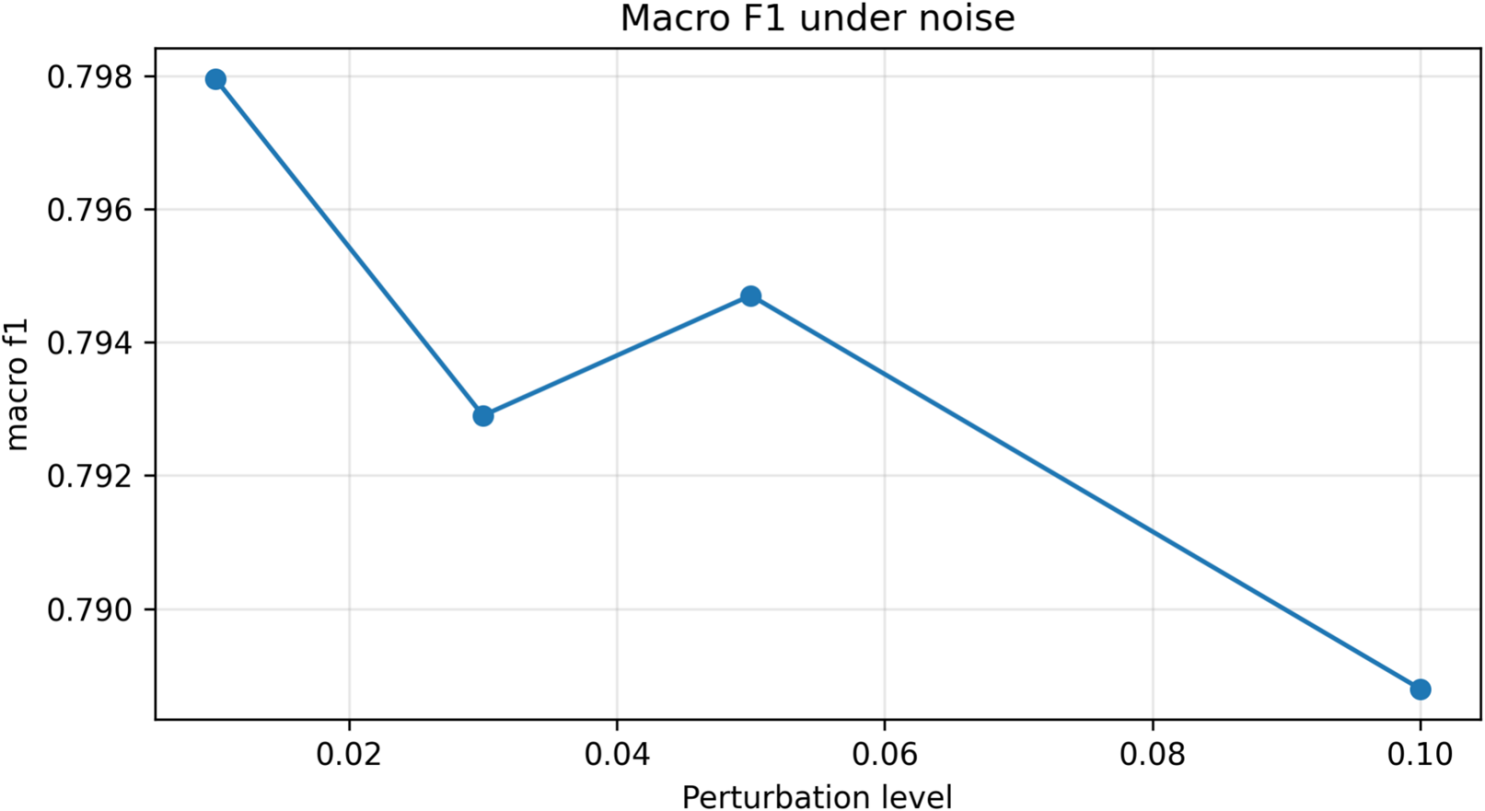
Macro-F1 of the tuned dynamic rough set model under increasing Gaussian feature noise.

**Fig. 8:**
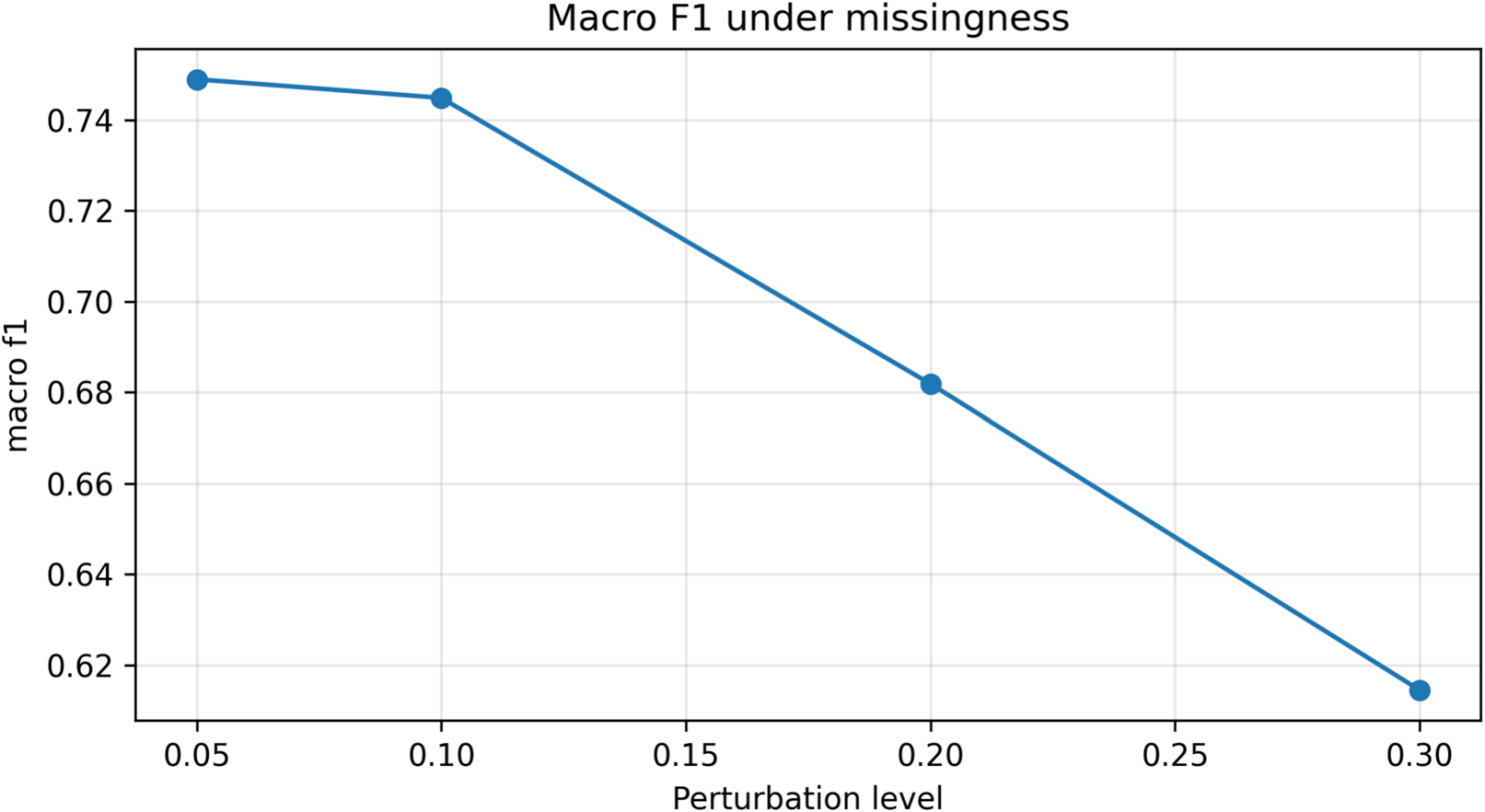
Macro-F1 of the tuned dynamic rough set model under increasing simulated missingness.

The effect of missingness was more pronounced. At missingness rate 0.30, Macro-F1 decreased to 0.6145, and the warning-class F1-score decreased to 0.2949. This suggests that the learned rough neighborhoods are robust to moderate feature perturbation, but sensitive to substantial missingness when many normalized feature values are replaced by imputed medians (Figs. 7, 8).

**Table 6:** Robustness of the tuned dynamic rough set model under feature noise and missingness on the C-MAPSS FD001 test set.

Perturbation	Level	Accuracy	Balanced accuracy	Macro-F1	Warning-class F1
Clean	0.0000	0.8647	0.7570	0.7895	0.5610
Noise	0.0100	0.8684	0.7670	0.7980	0.5732
Noise	0.0300	0.8665	0.7635	0.7929	0.5799
Noise	0.0500	0.8665	0.7662	0.7947	0.5799
Noise	0.1000	0.8553	0.7742	0.7888	0.5838
Missingness	0.0500	0.8421	0.7158	0.7490	0.5000
Missingness	0.1000	0.8421	0.7077	0.7449	0.4970
Missingness	0.2000	0.8102	0.6426	0.6819	0.3774
Missingness	0.3000	0.7801	0.5760	0.6145	0.2949

## Discussion

The proposed framework provides several interpretability advantages. First, the decision mechanism is based on explicit rough regions rather than only on posterior probabilities. A window can be inspected according to whether it belongs to a lower approximation, an upper approximation or a boundary region. Second, the support gap indicates whether the selected class is clearly dominant in the local neighborhood or only weakly preferred over another class. Third, the dynamic rough feature-reduction stage selects a compact subset of informative features. The selected features are ordinary sensor-derived statistics rather than latent features. This makes the model easier to inspect and supports the view that rough dependency can identify descriptors that preserve the decision structure.

Compared with recent deep sequential methods, the proposed framework has a different strength. Deep CNN, GRU, LSTM and transformer-style models can learn rich temporal representations and may outperform feature-based models when large labelled datasets are available. Their main disadvantage in industrial early-warning deployment is that the internal representation is often difficult to translate into certain, possible and ambiguous operating states. The proposed DRS model is less flexible as a universal function approximator, but it directly returns rough regions, support gaps and deferment information. Thus, it is most useful when interpretability and uncertainty qualification are operational requirements.

The robustness experiment also clarifies the practical behavior of the method. The model was stable under Gaussian feature noise, suggesting that the neighborhood structure is not overly sensitive to moderate perturbation of normalized features. By contrast, high missingness distorted the reference neighborhoods and reduced performance, especially for the warning class. Therefore, missingness-aware extensions are an important direction for future work.

### Practical implementation challenges

Several practical issues should be considered before using the framework in a real industrial monitoring environment. First, sensor streams must be synchronized and quality-controlled before windows are constructed. Second, normalization parameters and neighborhood radii should be calibrated on historical training data and periodically rechecked when operating conditions drift. Third, warning thresholds should be selected with engineers because a low threshold may create too many alarms, while a high threshold may miss useful early warnings. Fourth, deferred decisions require an operational response policy; for example, a deferred window may trigger additional inspection rather than an immediate shutdown. Finally, the computational cost of neighborhood search should be controlled in large-scale streaming systems by using approximate nearest-neighbor search, prototype selection or rolling reference sets.

## Limitations

The present study has several limitations. First, the empirical implementation was carried out on the C-MAPSS FD001 dataset only. Thus, the present experiments demonstrate the method on one industrial degradation benchmark. Although the mathematical construction may be transferred to other sequential monitoring settings, further validation on additional industrial and non-industrial datasets is needed before making broader empirical claims. In particular, FD002, FD003 and FD004 should be considered in future work because they introduce multiple operating conditions and/or multiple fault modes.

Second, the proposed method was compared numerically with classical machine-learning baselines, but not with a full set of large deep-learning time-series models. This choice was made to keep the comparison aligned with the feature-based rough-set representation. Future work should include additional numerical comparisons with one-dimensional convolutional networks, recurrent networks, CNN–GRU hybrids, transformer-style models and ROCKET-style methods.

Third, the current implementation uses a quantile-based neighborhood radius and hand-crafted window features. Although this makes the model interpretable and reproducible, performance may depend on the chosen window length, stride, feature set and validation grid. The sensitivity discussion in this paper therefore should be viewed as an initial analysis rather than a complete hyperparameter study.

Fourth, the warning output in this implementation is based on window-level class labels and rough local uncertainty. A prospective real-time early-warning evaluation with event-level alarm policies, alarm suppression rules, maintenance costs and false-alarm costs remains outside the scope of the present study.

Fifth, the present robustness analysis considers Gaussian feature noise and random missingness. Real industrial noise may be structured, sensor-specific, nonstationary or caused by calibration drift. Future work should therefore study more realistic missing-data mechanisms and sensor-fault scenarios.

## Conclusion

This paper introduced a dynamic rough set learning framework for uncertainty-aware early warning in industrial time-series systems. The proposed method transforms multivariate sensor trajectories into sliding-window decision systems, constructs time-dependent neighborhood rough approximations, and uses the resulting lower, upper and boundary regions to support interpretable warning decisions. A rough early-warning index was developed to quantify boundary-region expansion, and a dynamic dependency-based feature-reduction procedure was proposed to retain informative window descriptors.

The experimental study on the NASA C-MAPSS FD001 turbofan degradation dataset showed that the full dynamic rough set model achieved competitive classification performance while providing explicit uncertainty-aware outputs. Although Random Forest and XGBoost achieved stronger pure classification scores, the proposed method produced additional rough-set information that is useful for inspecting uncertain and transitional windows. Dynamic rough feature reduction reduced the feature set from 118 to 40 features while retaining reasonable performance. Robustness analysis showed stability under moderate Gaussian feature noise and sensitivity to substantial missingness.

The main practical contribution is that the framework gives maintenance analysts more than a class label. It identifies whether a window is supported by a certain rough region, belongs to a boundary region, has a small support gap or should be deferred for further inspection. This makes the method suitable for early-warning settings in which uncertainty itself is useful operational information.

Future research should address both theoretical and practical extensions. From the theoretical side, future work should study adaptive and data-driven neighborhood-radius selection, missingness-aware rough approximations, cost-sensitive rough support functions, class-balanced rough decision rules, and online temporal reducts for streaming data. From the practical side, the framework should be tested on the remaining C-MAPSS subsets and on additional industrial datasets with multiple operating conditions, multiple fault modes and nonstationary sensor behavior. Broader numerical comparisons with CNN, LSTM, GRU, transformer and ROCKET-style time-series models should also be conducted. Finally, future deployment studies should integrate event-level alarm policies, threshold recalibration, false-alarm cost analysis and engineer-in-the-loop inspection protocols.

Overall, the proposed framework contributes a mathematically grounded and reproducible method for dynamic rough uncertainty modeling in sequential decision systems. Its main value lies in connecting classification with interpretable rough regions, support gaps and deferred decisions, which are important for safety-critical industrial monitoring and other sequential decision systems.

## Data Availability

The data used in this study are publicly available. The FD001 subset was taken from the NASA C-MAPSS Jet Engine Simulated Data set, available through the NASA Open Data Portal: https://data.nasa.gov/dataset/cmapss-jet-engine-simulated-data. The compressed data file is available at: https://data.nasa.gov/docs/legacy/CMAPSSData.zip. No new experimental data were generated in this study. The processed window-level data can be reproduced from the public FD001 data using the preprocessing steps described in the manuscript and the accompanying code.

## List of symbols

*U*: Finite universe of objects in a static information system
*A*: Set of condition attributes or extracted features
*d*: Decision attribute or class label
$B\subseteq A$: Subset of features used to construct rough approximations
$B^*$: Selected feature subset obtained by dynamic rough feature reduction
*D* or $D_{c,t}$: Decision class; $D_{c,t}$ denotes class *c* at time/window index *t*
$U_t$: Dynamic universe of windows available at time/window index *t*
$DS_t$: Time-dependent sliding-window decision system
$\delta _{B,t}$: Dynamic dissimilarity induced by the feature subset *B*
$\rho _t$: Neighborhood radius at time/window index *t*
$q_\rho$: Quantile parameter used to select the adaptive neighborhood radius
${[x]}_{B,t}^{\rho _t}$: Dynamic neighborhood granule of object *x*
$\underline{R}_{B,t}^{\rho _t}(D)$: Dynamic lower approximation of decision class *D*
$\overline{R}_{B,t}^{\rho _t}(D)$: Dynamic upper approximation of decision class *D*
$BND_{B,t}^{\rho _t}(D)$: Dynamic boundary region of decision class *D*
$E_{B,t}^{\rho _t}(D)$: Rough early-warning index of decision class *D*
$\Gamma _B(d)$: average dynamic dependency degree of *B* with respect to *d*
*h*: Sliding-window length
*s*: Sliding-window stride
$\Delta$: Warning horizon used to define warning labels
$\theta _G$: Support-gap threshold for accepting or deferring a decision
$\theta _E$: Local uncertainty threshold for warning generation
$\pi ^R_{c,t}(x)$: Reference-neighborhood rough support of class *c* for test window *x*
$U^\textrm{loc}_t(x)$: Local uncertainty score of test window *x*

## Code availability

The complete Python implementation used to generate the experimental results, tables and figures reported in this study is publicly available in the Zenodo repository at https://doi.org/10.5281/zenodo.21173325. The archived software includes the code for data preprocessing, sliding-window construction, feature extraction, training–validation–test splitting, training-only normalization, dynamic rough-set modelling, dynamic rough feature reduction, baseline comparisons, robustness analysis, and generation of the reported tables and figures. The repository also contains the software dependencies, parameter settings, processed experimental data, generated results, and instructions required to reproduce the computational experiments. The raw NASA C-MAPSS data are not redistributed; instructions for obtaining and placing the required raw data are included in the archive.
